# Trans fatty acids alter renal health in TGFβ3 mice with fibrosis revealed by metabolomics and lipidomics

**DOI:** 10.1016/j.isci.2026.116506

**Published:** 2026-06-30

**Authors:** Borja Lanzon, Elia Escasany, Almudena G. Carrasco, Carolina Gonzalez-Riano, Daniel Horrillo, Patricia Corrales, Antonia García, Francisco J. Ruperez, Adriana Izquierdo-Lahuerta, Gema Medina-Gomez

**Affiliations:** 1 Departamento de Ciencias Básicas de La Salud, Facultad de Ciencias de La Salud, Universidad Rey Juan Carlos, Madrid, Spain; 2 Centro de Metabolómica y Bioanálisis (CEMBIO), Facultad de Farmacia, Universidad San Pablo-CEU, CEU Universities, Urbanización Montepríncipe, Boadilla Del Monte, Madrid, Spain; 3 LAFEMEX Laboratory, Área de Bioquímica y Biología Molecular, Departamento de Ciencias Básicas de La Salud, Facultad de Ciencias de La Salud, Universidad Rey Juan Carlos, Avda. de Atenas s/n. 28922-Alcorcón, Madrid, Spain

**Keywords:** trans-fatty acids, renal fibrosis, TGFβ3 deficiency, lipid droplet accumulation, lipidomics, metabolomics

## Abstract

*Trans*-fatty acids, abundant in Western diets, are detrimental to kidney health, yet their impact on renal metabolic and lipid homeostasis remains poorly understood. Here, we examine the effects of a *trans*-fat diet on renal function using wild-type, and mice heterozygous for transforming growth factor β3 (Tgfβ3) deletion that present kidney damage and fibrosis. Using integrated metabolomic, lipidomic, molecular, and cellular analyses, we show that in wild-type mice, the *trans*-fat diet disrupted renal lipid metabolism, characterized by a marked depletion of serum triglycerides, alterations in lipid-synthesis genes in the kidney, and metabolic signatures of mitochondrial dysfunction. In Tgfβ3-heterozygous kidneys, the *trans*-fat diet exacerbated oxidative stress and was associated with severe depletion of lipid species and aggravated podocyte loss. Notably, the combination of *trans*-fat consumption and Tgfβ3 deficiency caused intracellular accumulation of enlarged lipid droplets and taurine depletion in the kidneys. Together, a *trans*-fat-rich diet markedly intensifies the renal injury associated with Tgfβ3 deficiency.

## Highlights

• Trans fats disrupt renal lipid metabolism
• Trans fats deplete triglycerides and impair mitochondrial function
• Trans fats and TGFβ3 deficiency promote enlarged renal lipid droplets
• Core lipid signature marks renal impairment independent of disease origin

## Graphical abstract

**Figure:**
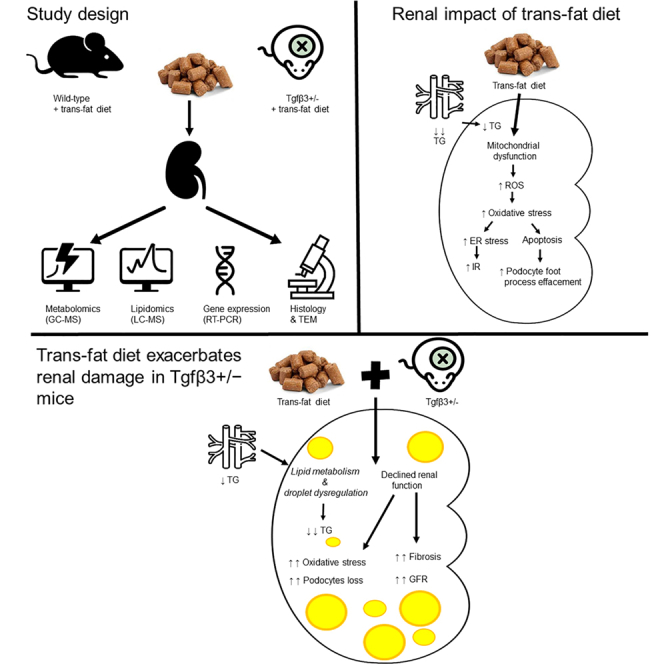

## Teaser

Pathophysiology; Lipidomics; Metabolomics

## Introduction

Obesity has emerged as a growing global health crisis that requires urgent need for effective prevention strategies and treatments.^1^ Understanding its complex pathophysiology and associated complications requires robust research methodologies, including the use of animal models that mimic the characteristics of human obesity and its comorbidities.^2^^,^^3^

Epidemiological studies associate high-fat diets with obesity, a relationship replicated in rodent models. High-fat diets (HFDs), such as the “cafeteria diet,”^4^ which mimics the western human diets, induce obesity by rapidly impairing insulin and leptin.^4^ *Trans*-fats of vegetable origin, a less explored alternative in research but commonly consumed, are primarily produced during the hydrogenation of vegetable oils and in ruminants.^5^ These fats have distinct effects and should not be grouped as a single entity.^6^ *Trans*-fats increase cardiovascular risk and are linked to diabetes, inflammation, and cancer,^7^ prompting the WHO to advise minimizing their intake.^7^

Obesity is a risk factor for renal damage, as compensatory hyperfiltration occurs to accommodate the increased metabolic demand caused by weight gain. This increased glomerular pressure can lead to kidney damage and a higher risk of chronic kidney disease (CKD).^8^ Obesity also enhances the endocrine activity of adipose tissue, releasing adipokines involved in insulin resistance, inflammation, and oxidative stress in the kidney.^9^ These adipokines stimulate the expression of profibrotic molecules, such as transforming growth factor β (Tgfβ), playing a pivotal role in the development of renal fibrosis.^8^

TGFβ is a family of cytokines that comprises multifunctional proteins involved in processes such as cell differentiation and fibrosis. In mammals, three isoforms have been described (TGFβ1, TGFβ2, and TGFβ3), which share a high degree of homology and were initially presumed to be functionally redundant.^10^ However, functional studies using deleterious forms of these isoforms demonstrated that they exert distinct biological roles. TGFβ1 is the most extensively studied isoform and is well known for its profibrotic role.^11^ In previous studies, we identified TGFβ3 as a critical regulator of adipocyte proliferation in adipose tissue.^12^ TGFβ3 is often described as a paralog of TGFβ1, but published results on the function of TGFβ3 are highly varied and controversial, with some studies suggesting an antifibrotic function^13^^,^^14^ and others supporting the view that its activity is redundant to that of TGFβ1, i.e., profibrotic.^15^ At the topical level, it has been reported that the application of TGFβ3 to wounds has antifibrotic effects, unlike TGFβ1 and TGFβ2.^16^^,^^17^ However, other studies suggest that TGFβ3 has additive effects to TGFβ1 in mesangial and tubular cells (Yu et al., 2003). Most of these studies were conducted *in vitro*, and TGFβ3 was added exogenously to the cells, more physiological experiments are needed to clarify this controversy.

In this study, we investigated the metabolic, histological, and metabolomic alterations induced by a *trans*-fat diet (TFD) of vegetable origin in the kidneys of wild-type animals. In parallel, we evaluated the effects of this diet in a murine model with partial deletion of Tgfβ3, previously characterized by renal fibrosis, dysregulated lipid metabolism, oxidative stress, and mitochondrial dysfunction.^18^ By comparing these models, this work aims to define kidney-specific alterations driven by *trans*-fat consumption in healthy mice and in mice with pre-existing renal damage, extending previous studies that have primarily focused on high-fat diets of animal origin.^19^

## Results

### *Tgfβ3* expression is downregulated in a lipotoxic model *in vitro* and in obese mice

**Figure 1:**
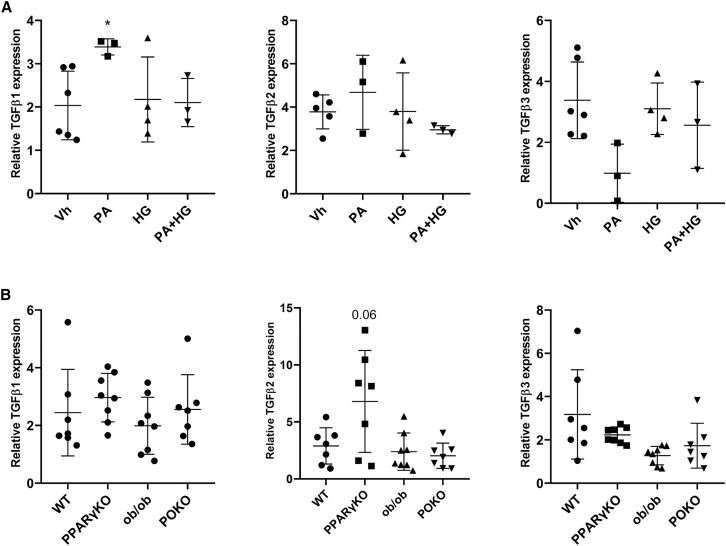
TGFβ3 Expression decreases in podocytes with lipids and in the whole kidneys of other lipotoxic models (A) Relative mRNA expression of TGFβ3, TGFβ1, and TGFβ2 (*n* = 3–6) in podocytes treated for 24 h under different hypercaloric conditions: PA (500 μM) and HG (25 mM). Data are expressed as mean ± SEM. Vh, vehicle; PA, palmitic acid; HG, high glucose. (B) Relative mRNA expression of TGFβ3, TGFβ1, and TGFβ2 (*n* = 7–8) in whole kidneys from wild-type (WT), PPARγ2KO, ob/ob, and POKO mice. PPARγ2KO, peroxisome proliferator-activated receptor gamma 2 knockout; ob/ob, leptin-deficient mice; POKO, PPARγ2 knockout ob/ob mice. Data are expressed as mean ± SEM. ∗*p* < 0.05 for WT and Vh vs. other conditions in (A) and (B). Statistical analyses were performed as described in the Methods section. Exact *p* values and statistical tests are indicated when applicable.

To assess the role of Tgfβ3 in renal lipotoxicity, we measured its expression in podocytes treated with palmitic acid (PA) and high glucose (HG), as well as in kidney samples from ob/ob, POKO, and PPARγ2 knockout mice. *Tgfβ1* mRNA levels were significantly upregulated in podocytes treated with PA, while *Tgfβ2* showed no changes. In contrast, *Tgfβ3* was significantly downregulated (Figure 1A). Similarly, Tgfβ3 levels were significantly reduced in the kidneys of ob/ob mice relative to lean controls (Figure 1B).

### *Trans*-fat diet induces renal damage despite triglyceride depletion, with distinct endpoints during fibrosis

The effects of TFD were evaluated in both wild-type (Tgfβ3^+/+^) and heterozygous Tgfβ3 mice (Tgfβ3^+/−^), a murine model of fibrosis previously characterized on a control diet (CD).^18^

**Figure 2:**
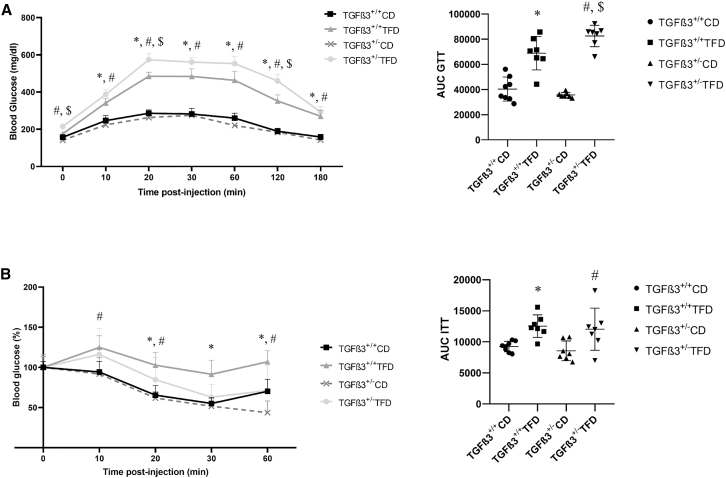
Effects of *trans*-fat diet and Tgfβ3 deletion on Glucose and Insulin tolerance in mice (A) Response curves from *in vivo* glucose tolerance tests (GTT) and (B) insulin tolerance tests (ITT) were analyzed for Tgfβ3^+/+^ CD, Tgfβ3^+/+^ TFD, Tgfβ3^+/−^ CD, and Tgfβ3^+/−^ TFD mice (*n* = 7–8). All data are expressed as mean ± SEM. ∗*p* < 0.05. AUC, area under the curve. (¥) Tgfβ3^+/−^ CD vs. Tgfβ3^+/+^ CD; (∗) Tgfβ3^+/+^ TFD vs. Tgfβ3^+/+^ CD; (#) Tgfβ3^+/−^ TFD vs. Tgfβ3^+/−^ CD; ($) Tgfβ3^+/−^ TFD vs. Tgfβ3^+/+^ TFD.

In male Tgfβ3^+/+^ mice, TFD led to significant increases in body weight, low-density lipoprotein (LDL) and high-density lipoprotein (HDL) cholesterol levels; however, serum triglycerides (TGs) were unexpectedly decreased, without changes in glucose or NEFA levels when compared with control mice fed CD. TFD also produced a significant increase in glomerular filtration rate (GFR), albumin/creatinine ratio (ACR), and reduced urine volume in Tgfβ3^+/+^ TFD relative to control mice on CD. Additionally, Tgfβ3^+/+^ TFD mice were more glucose intolerant and insulin-resistant than Tgfβ3^+/+^ CD controls (Table 1; Figures 2A and 2B).

**Table 1:** Metabolic and biochemical measurements of Tgfβ3^+/+^ and Tgfβ3^+/−^ mice on CD and TFD at 16 weeks of age

	TGFΒ3^+/+^ CD	TGFΒ3^+/+^ TFD	TGFΒ3^+/−^ CD	TGFΒ3^+/−^ TFD
**Body weight (g)**	26,2 ± 0,6	31,73 ± 0,32 ∗	26,4 ± 0,40	31,6 ± 0,32 #
**Kidney weight (g)**	0,29 ± 0.012	0,29 ± 0,01	0,30 ± 0.011	0,32 ± 0,01
**Serum glucose (mmol/L)**	12,23 ± 0,86	9,62 ± 0,82	10,63 ± 0,85	10,66 ± 0,83
**Serum triglycerides (mg/dL)**	210,33 ± 17,55	93,77 ± 11,12 ∗	207 ± 0,10	114,30 ± 7,74 #
**Total cholesterol (mg/dL)**	140,90 ± 6,12	171,40 ± 21,46 ∗	146,15 ± 6,05	187,64 ± 18,98 #
**LDL (mmol/L)**	0,55 ± 0,01	0,79 ± 0,18 ∗	0,67 ± 0,17	1,06 ± 0,24 #
**HDL (mmol/L)**	2,66 ± 0,23	3,57 ± 0,41 ∗	2,72 ± 0,13	3,53 ± 0,32 #
**NEFAs (mmol/L)**	3,82 ± 0,04	2,78 ± 0,6	4,10 ± 0,80	3,26 ± 0,2
**Serum albumin (g/L)**	4,58 ± 0,11	4,71 ± 0,23	4,45 ± 0,13	4,82 ± 0,24
**GFR (μL/min)**	181,44 ± 54,59	254,21 ± 72,99 ∗	145,62 ± 58,78	372,83 ± 79,69 #, $
**ACR (μg/μmol)**	4,90 ± 0,62	16,05 ± 2,67 ∗	9,37 ± 2,19	23,07 ± 5,46 #
**Urine volume (μL)**	732 ± 112,55	335 ± 51,45 ∗	435 ± 83,33 ¥	368 ± 36,34

Data are expressed as mean ± SEM (*n* = 7–12). LDL, low-density lipoprotein; HDL, high-density lipoprotein; NEFAs, non-esterified fatty acids; GFR, glomerular filtration rate; ACR, albumin creatinine ratio. Metabolic and biochemical parameters were considered significant when a *p* value ≤ 0.05 was obtained. (¥) Tgfβ3^+/−^ CD vs. Tgfβ3^+/+^ CD; (∗) Tgfβ3^+/+^ TFD vs. Tgfβ3^+/+^ CD; (#) Tgfβ3^+/−^ TFD vs. Tgfβ3^+/−^ CD; ($) Tgfβ3^+/−^ TFD vs. Tgfβ3^+/+^ TFD. Table modified from.^18^

Heterozygous Tgfβ3^+/−^ mice on TFD exhibited similar significant alterations to those observed in wild-type mice when compared with their respective CD-fed controls (Table 1; Figures 2A and 2B).

However, direct comparison between Tgfβ3^+/−^ and wild-type mice under TFD revealed a significantly higher GFR in mice with partial Tgfβ3 deletion (Table 1). This renal alteration was not accompanied by significant differences in body weight, cholesterol levels or ACR between the two groups (Table 1). Furthermore, Tgfβ3^+/−^ TFD mice were more glucose intolerant, but not more insulin resistant than the Tgfβ3^+/+^ TFD group (Figures 2A and 2B).

**Figure 3:**
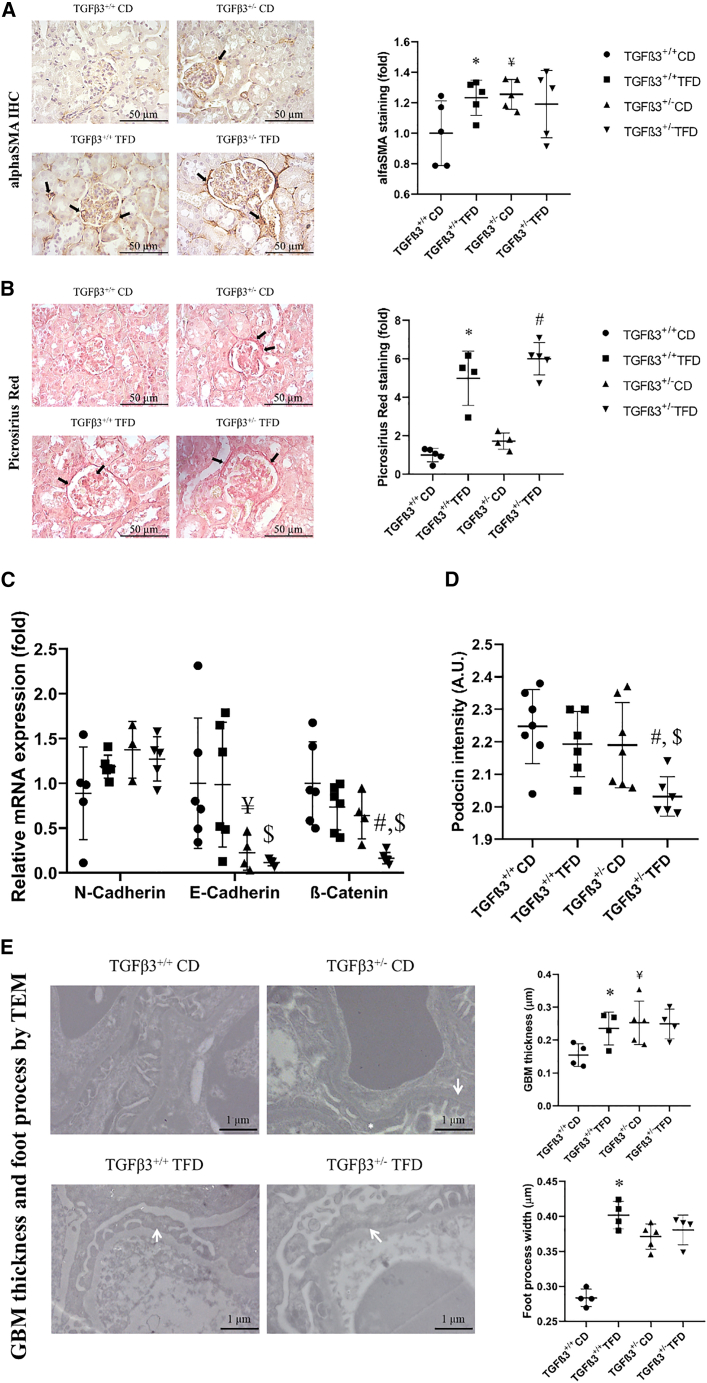
Fibrosis, epithelial-mesenchymal transition (EMT), and transmission electron microscopy tests in whole kidneys of Tgfβ3^+/+^ CD, Tgfβ3^+/+^ TFD, Tgfβ3^+/−^ CD, and Tgfβ3^+/−^ TFD mice (A) α-SMA immunohistochemistry (*n* = 5) and (B), Picrosirius Red staining (*n* = 4–6) in 4-month-old mice. Scale bars, 50 μm. (C) Relative mRNA expression of N-cadherin, E-cadherin and β-catenin in whole kidney of 4-month-old mice (*n* = 5–6). (D) Quantification of podocytes by flow cytometry using the podocin marker in whole kidneys. (E) GBM thickness and foot process width measured on transmission electron microscopy images in the whole kidney of 4-month-old mice (*n* = 4–5). GBM, glomerular basement membrane. Optical microscope images were taken at a magnification of 400× and TEM images at 20k. Black arrows mark positively stained areas of the tissue. TEM, transmission electron microscopy. White arrows mark thickened areas of the GBM, and white asterisks mark foot process effacement. (A), (B) and (C) data are expressed as mean ± SEM. Parameters were considered significant when a *p* value ≤0.05 was obtained. (¥) Tgfβ3^+/−^ CD vs. Tgfβ3^+/+^ CD; (∗) Tgfβ3^+/+^ TFD vs. Tgfβ3^+/+^ CD; (#) Tgfβ3^+/−^ TFD vs. Tgfβ3^+/−^ CD; ($) Tgfβ3^+/−^ TFD vs. Tgfβ3^+/+^ TFD.

To analyze the profibrotic effects of the TFD in the kidney, immunohistochemical (IHC) staining for α-smooth muscle actin (α-SMA) and Picrosirius Red staining were performed in wild-type animals (Figures 3A and 3B). TFD induced significantly higher levels of fibroblasts and collagen production in Tgfβ3^+/+^ mice. However, TFD did not cause alterations in the expression of epithelial-mesenchymal transition (EMT) genes (E-cadherin, β-catenin, and N-cadherin) or in the number of podocytes in the kidneys of wild-type animals relative to controls on CD (Figures 3C and 3D). Electron microscopy analysis revealed marked structural changes in the glomeruli of TFD wild-type animals, including significant thickening of the glomerular basement membrane (GBM) and podocyte foot process effacement (FPE) (Figure 3E).

On the other hand, partial deletion of Tgfβ3^+/−^ in TFD animals caused a significant increase in Picrosirius Red staining (Figure 3B) and a significant decrease in the β-catenin EMT marker relative to the Tgfβ3^+/−^ CD group (Figure 3C). Despite the absence of detectable structural differences in podocyte morphology (Figure 3E), there was a significant reduction in the number of podocytes in the kidneys of the Tgfβ3^+/−^ TFD mice relative to the Tgfβ3^+/−^ CD group (Figure 3D).

Although Tgfβ3^+/−^ TFD animals did not show worse fibrosis or major differences in podocyte foot process width when compared with Tgfβ3^+/+^ TFD animals (Figures 3A, 3B, and 3E), Tgfβ3^+/−^ TFD mice exhibited a significant reduction in EMT E-cadherin and β-catenin genes. These mice also showed a decreased number of podocytes in their kidneys relative to their Tgfβ3^+/+^ controls fed the same diet (Figures 3C and 3D).

### Tgfβ3 deficiency promotes the accumulation of enlarged lipid droplets in the kidneys of *trans*-fat-fed mice

**Figure 4:**
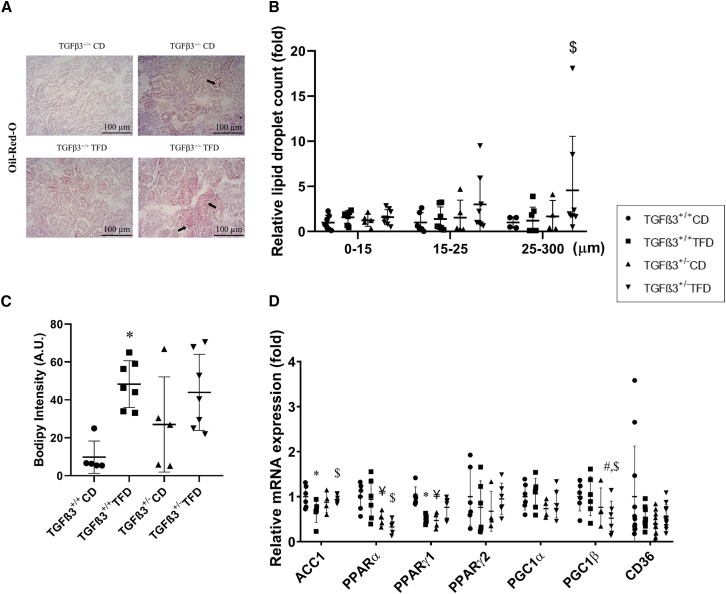
Renal lipid metabolism in whole kidneys of Tgfβ3^+/+^ CD, Tgfβ3^+/+^ TFD, Tgfβ3^+/−^ CD, and Tgfβ3^+/−^ TFD mice (A) Oil Red O staining in the kidney at 200× magnification. Scale bars, 100 μm. (B) Quantification of lipid droplet accumulation in the kidney from Oil-Red technique in whole kidney (*n* = 5–8). The quantification is presented across different size ranges (0–15, 15–25, and 25–300 μm). Results are expressed as relative lipid droplet count (fold) compared to the control condition. (C) Bodipy staining intensity measured by flow cytometry (*n* = 5–7). (D) Relative mRNA expression of lipid metabolism genes in whole kidney (*n* = 5–7). Black arrows mark red dots representing lipid droplets. Data are shown as mean ± SEM. Parameters were considered significant when a *p* value ≤0.05 was obtained. (¥) Tgfβ3^+/−^ CD vs. Tgfβ3^+/+^ CD; (∗) Tgfβ3^+/+^ TFD vs. Tgfβ3^+/+^ CD; (#) Tgfβ3^+/−^ TFD vs. Tgfβ3^+/−^ CD; ($) Tgfβ3^+/−^ TFD vs. Tgfβ3^+/+^ TFD.

Wild-type mice fed TFD showed increased lipid accumulation in the kidneys (Figures 4A and 4C). However, no significant changes in the number of lipid droplets were observed in the kidneys of Tgfβ3^+/+^ TFD compared to Tgfβ3^+/+^ CD (Figure 4B), although a trend toward higher lipid accumulation was detected in the TFD group. This was accompanied by a significant reduction in the expression of key genes involved in lipid synthesis (ACC and PPARγ) between these groups (Figure 4D).

The kidneys of Tgfβ3^+/−^ TFD animals exhibited increased neutral lipid accumulation, as assessed by Oil Red O staining, a greater number of lipid droplets, and a significant reduction in lipid oxidation processes (PGC1β) when compared with Tgfβ3^+/−^ CD mice (Figures 4A, 4B, 4C, and 4D).

Although Tgfβ3^+/−^ TFD and Tgfβ3^+/+^ TFD kidneys exhibited comparable levels of lipid accumulation (Figures 4A and 4C), Tgfβ3^**+/−**^ TFD animals showed a significantly greater number of lipid droplets (Figure 4B). A similar pattern was observed between Tgfβ3^+/−^ CD and Tgfβ3^+/+^ CD mice (Figure 4B), albeit to a lesser extent. In addition, Tgfβ3^+/−^ TFD mice displayed a significant upregulation of ACC1, along with a marked reduction in PPARα and PGC1β relative to Tgfβ3^+/+^ TFD mice (Figure 4D).

### Tgfβ3 deletion does not exacerbate mitochondrial alterations or reactive oxygen species levels induced by a *trans*-fat diet

**Figure 5:**
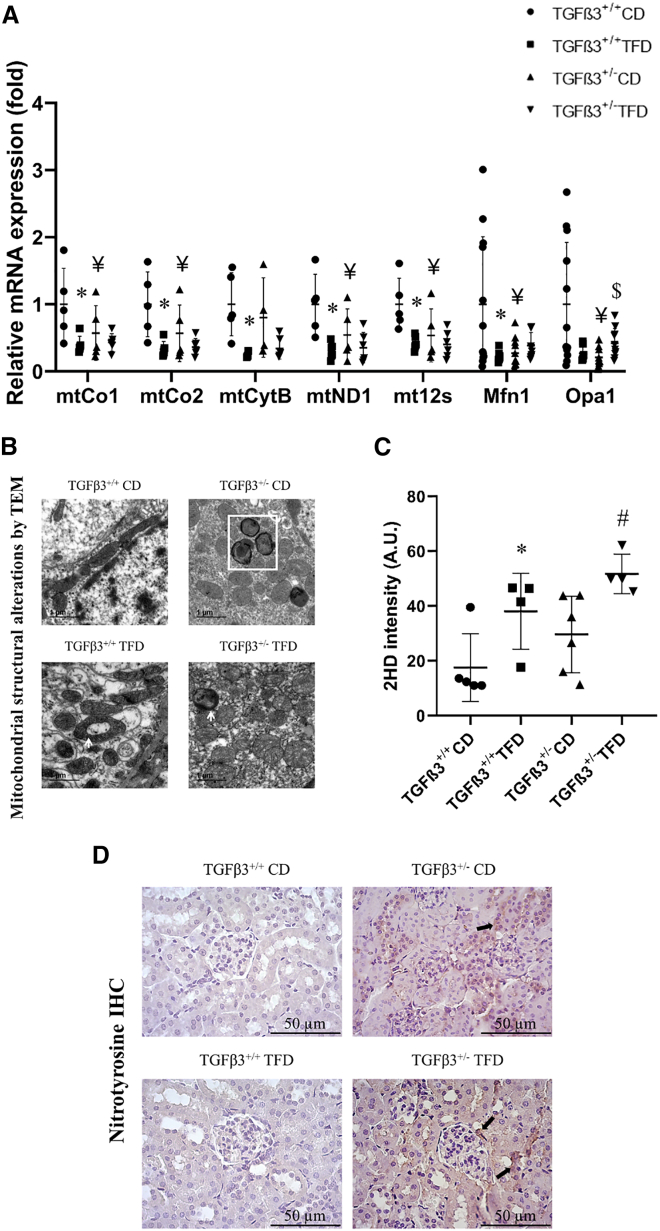
Mitochondrial alterations in whole kidneys of Tgfβ3^+/+^ CD, Tgfβ3^+/+^ TFD, Tgfβ3^+/−^ CD, and Tgfβ3^+/−^ TFD mice (A) Relative mRNA expression of mitochondrial and mitochondria-related genes (*n* 5–6). (B) Mitochondrial structural alterations by transmission electron microscopy. Onion-like shaped mitochondria is squared in white in the picture. White arrows indicate abnormal structures. Scale bars, 1 μm. (C) Dihydroethidium staining intensity measured by flow cytometry (*n* = 4–5). (D) Nitrotyrosine immunohistochemistry (*n* = 6). Scale bars, 50 μm. Optical microscope images were taken at a magnification of 400× and TEM images at 20k. (A) Data are shown as mean ± SEM. Parameters were considered significant when a *p* value ≤0.05 was obtained. (¥) Tgfβ3^+/−^ CD vs. Tgfβ3^+/+^ CD; (∗) Tgfβ3^+/+^ TFD vs. Tgfβ3^+/+^ CD; (#) Tgfβ3^+/−^ TFD vs. Tgfβ3^+/−^ CD; ($) Tgfβ3^+/−^ TFD vs. Tgfβ3^+/+^ TFD. A.U., arbitrary units; IHC, immunohistochemistry; Mfn1, mitochondrial fusion protein type 1; mtCo1, cytochrome oxidase subunit 1; mtCo2, cytochrome oxidase subunit 2; mtCytB, cytochrome *b*; mtND1, NADH dehydrogenase subunit 1; mt12S, mitochondrial 12S ribosomal gene; OPA1, mitochondrial dynamin such as GTPase; TEM, transmission electron microscopy; 2HD, dihydroethidium.

Gene expression analysis related to mitochondrial metabolism revealed a significant reduction in mtCo1, mtCo2, mtCytb, mtND1, mt12s, and Mfn1 in wild-type mice fed a TFD compared with CD control animals (Figure 5A). Structural analysis via electron microscopy revealed pathological mitochondrial structures in Tgfβ3^+/+^ TFD mice, including “donut” and “onion”-shaped mitochondria (Figure 5B). In addition, Tgfβ3^+/+^ TFD mice exhibited a significant accumulation of reactive oxygen species relative to Tgfβ3^+/+^ CD mice (Figure 5C).

No significant differences in mitochondrial gene expression were detected between Tgfβ3^+/−^ TFD and Tgfβ3^+/−^ CD mice (Figure 5A), as similar alterations were already present under CD conditions. Similar pathological mitochondrial structures were observed in Tgfβ3^+/−^ TFD and Tgfβ3^+/−^ CD mice (Figure 5B). However, kidneys of Tgfβ3^+/−^ TFD mice exhibited a significant increase in ROS relative to their heterozygous controls (Figures 5C and 5D).

Tgfβ3^+/−^ TFD mice revealed a significant increase in Opa1 mRNA compared with Tgfβ3^+/+^ TFD animals (Figure 5A). Despite this difference, both genotypes exhibited aberrant mitochondrial structures and comparable oxidative stress levels under TFD conditions (Figures 5B, 5C, and 5D).

### Consistent triglyceride depletion and core lipid signature are linked to renal impairment under *trans*-fat diet and TGFβ3 deficiency

**Figure 6:**
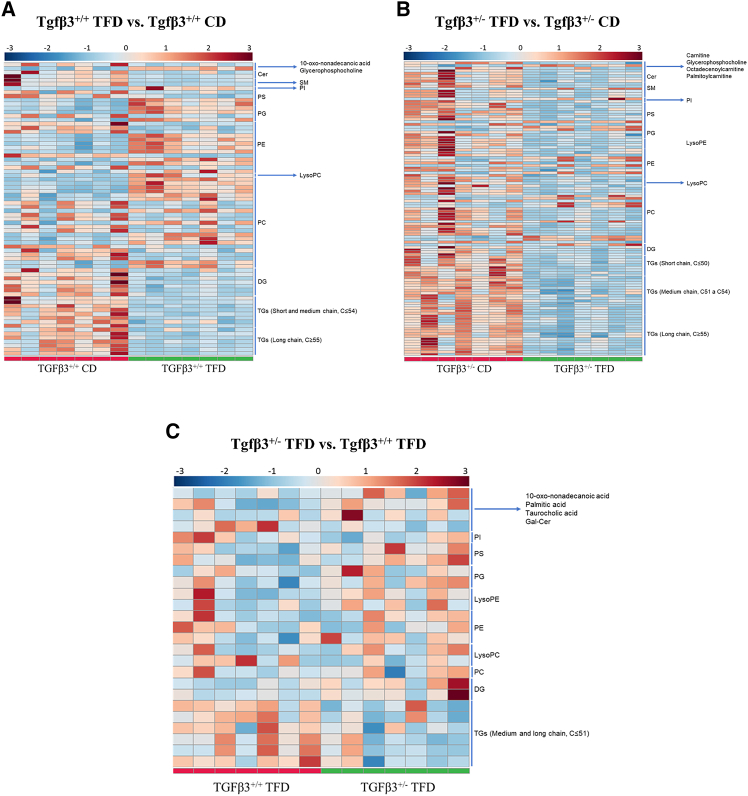
Heatmap of lipid distribution in the kidneys under different experimental conditions (A) Tgfβ3^+/+^ TFD vs. Tgfβ3^+/+^ CD. Animals from the Tgfβ3^+/+^ CD group (left) are shown in red, and animals from the Tgfβ3^+/+^ TFD group (right) are represented in green. (B) Tgfβ3^+/-^ TFD vs. Tgfβ3^+/-^ CD. Animals from the Tgfβ3^+/-^ CD group (left) are shown in red, and animals from the Tgfβ3^+/-^ TFD group (right) are represented in green. (C) Tgfβ3^+/-^ TFD vs. Tgfβ3^+/+^ TFD. Animals from the Tgfβ3^+/+^ TFD group (left) are shown in red, and animals from the Tgfβ3^+/-^ TFD group (right) are represented in green. The heatmap represents the intensity of significantly different compounds. All experiments were performed in whole kidneys of 4-month-old male mice (*n* = 7). TG, triglycerides; DG, diglycerides; PC, phosphatidylcholines; LysoPC, lysophosphatidylcholines; PE, phosphatidylethanolamines; PG, phosphatidylglycerols; PS, phosphatidylserines; PI, phosphatidylinositols; SM, sphingomyelins; Cer, ceramides.

Lipidomic analysis of Tgfβ3^+/+^ TFD mice relative to the Tgfβ3^+/+^ CD animals using liquid chromatography-mass spectrometry (LC-MS) revealed a total of 40 significantly altered lipid species (Figures 6 and S2). TFD promoted a significant decrease in all detected TGs and diglycerides (DGs), with 15 TGs and 7 DGs significantly reduced in the Tgfβ3^+/+^ TFD mice. In addition, 23 phosphatidylcholines (PCs) and 13 phosphatidylethanolamines (PEs) were significantly altered, although the direction of the changes did not follow a consistent trend. Additionally, 17 plasmalogens were identified, of which 13 were upregulated in the Tgfβ3^+/+^ TFD group.

In contrast, *trans*-fat feeding in mice with the partial deletion of Tgfβ3 resulted in 124 significantly altered lipid species. Most of these lipid species were downregulated in the Tgfβ3^+/−^ TFD group versus the Tgfβ3^+/−^ CD group (Figures 6 and S3). Specifically, 39 significant TGs, 15 PCs, 9 PEs, 3 lysophosphatidylcholines (LysoPCs), 4 lysophosphatidylethanolamines (LysoPEs), 6 phosphatidylserines (PSs), and 12 plasmalogens were downregulated in the Tgfβ3^+/−^ TFD mice compared to the Tgfβ3^+/−^ CD mice.

Direct comparison between Tgfβ3^+/−^ TFD and Tgfβ3^+/+^ TFD mice identified 25 significantly altered lipid species (Figures 6 and S4). This lipidome between these animals was more restricted than in the other comparisons, with TGs being the most relevant subclass (25% of the total) and fewer compounds detected among other lipid classes (Figures 6 and S4). All significant TGs were downregulated, and 2 DGs were upregulated in Tgfβ3^+/−^ TFD animals relative to the Tgfβ3^+/+^ TFD group.

Venn diagram analysis enabled the identification of lipid species associated with the *trans*-fat feeding, Tgfβ3 deletion, or both conditions (Figure S1). Four lipid species were common to all three comparisons (PS(P-40:4), PC(32:1), TG(51:1), TG(53:1)), potentially representing a core lipid signature associated with renal impairment. A total of 47 lipid species were shared between the Tgfβ3^+/+^ TFD vs. Tgfβ3^+/+^ CD and Tgfβ3^+/−^ TFD vs. Tgfβ3^+/−^ CD groups, suggesting a common lipid response to the TFD regardless of genotype.

In addition, four exclusive lipid species—PG(39:1), PE(P-36:1), PE(36:3), and PE(O-36:4)—were detected only between Tgfβ3^+/+^ TFD vs. Tgfβ3^+/+^ CD mice. In contrast, the Tgfβ3^+/−^ TFD vs. Tgfβ3^+/−^ CD dataset uniquely identified 23 downregulated TG species. Moreover, 14 lipid species were specific to the Tgfβ3^+/−^ TFD vs. Tgfβ3^+/+^ TFD condition, indicating lipid alterations specifically associated with Tgfβ3 deletion in the obesogenic context (Figure S1).

**Figure 7:**
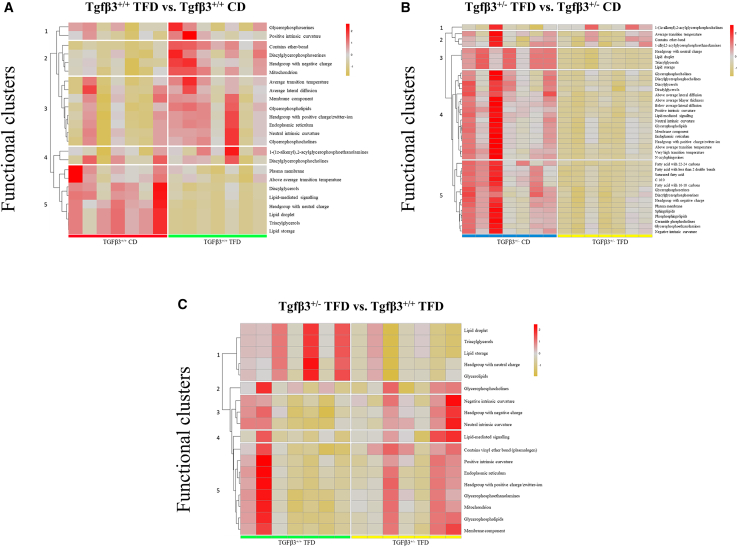
Heatmap of significant lipids identified under different experimental conditions generated using LION (A) Tgfβ3^+/+^ TFD vs. Tgfβ3^+/+^ CD. Samples from the Tgfβ3^+/+^ TFD group are represented in green, and samples from the Tgfβ3^+/+^ CD group are shown in red. (B) Tgfβ3^+/-^ TFD vs. Tgfβ3^+/-^ CD. Samples from the Tgfβ3^+/-^ TFD group are shown in yellow, and samples from the Tgfβ3^+/-^ CD group are shown in blue. (C) Tgfβ3^+/-^ TFD vs. Tgfβ3^+/+^ TFD. Samples from the Tgfβ3^+/-^ TFD group are shown in yellow, and samples from the Tgfβ3^+/+^ TFD group are shown in green. The colors in the heatmap represent increased (red) or decreased (yellow) LION terms. The heatmap displays LION terms on the left and the dendrogram of LION term clustering on the right. All experiments were performed in whole kidneys of 4-month-old male mice (*n* = 7). The number of clusters is indicated numerically in the heatmap. LION, Lipid Ontology

LION enrichment analysis grouped significantly altered lipids from each comparison into functional clusters based on their biological or cellular roles (Figure 7). In wild-type mice fed the TFD, increased enrichment of mitochondria-associated lipid terms was observed (cluster 2), while terms related to lipid storage were reduced (cluster 5) (Figure 7).

In heterozygous Tgfβ3^+/−^ mice, TFD led to a decrease in lipid terms associated with storage lipids (cluster 3), signaling lipids and membrane components (cluster 4), as well as saturated fatty acids (SFAs) and phospholipids (cluster 5), relative to CD animals. Moreover, lipid distribution across these clusters appeared more homogeneous in Tgfβ3^+/−^ TFD mice than in their control counterparts (Figure 7).

Finally, comparison between Tgfβ3^+/−^ and wild-type mice under the TFD revealed a decrease in lipid storage-related terms (cluster 1) in the Tgfβ3^+/−^ TFD group, whereas no consistent trend was observed across the remaining clusters (Figure 7).

### Tgfβ3 deletion leads to a downregulation of essential and non-essential amino acids and taurine, independent of diet

**Figure 8:**
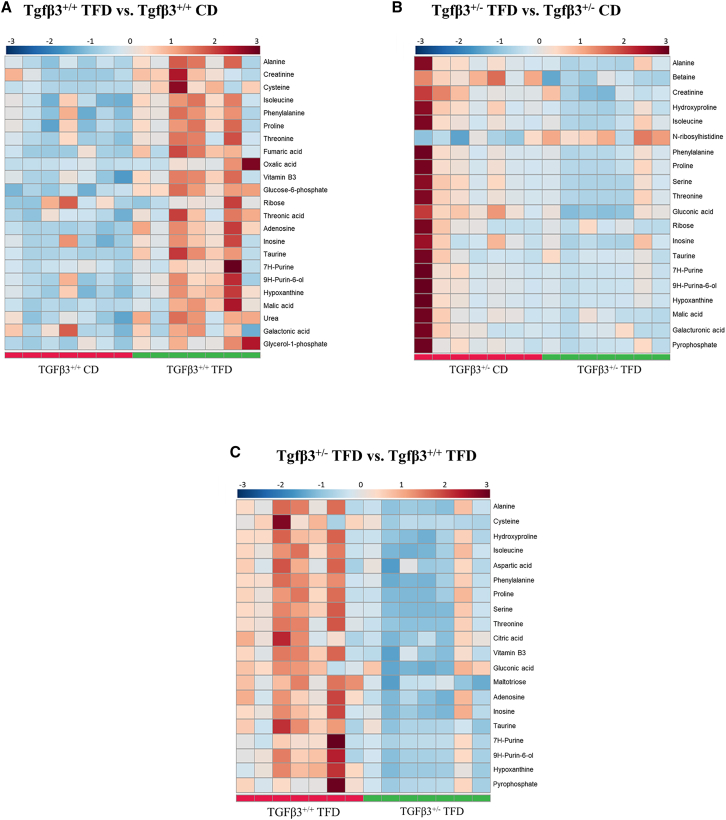
Heatmap of the distribution of polar compounds in the kidneys under different experimental conditions (A) Tgfβ3^+/+^ TFD vs. Tgfβ3^+/+^ CD. Animals from the Tgfβ3^+/+^ CD group (left) are shown in red, and animals from the Tgfβ3^+/+^ TFD group (right) are shown in green. (B) Tgfβ3^+/-^ TFD vs. Tgfβ3^+/-^ CD. Animals from the Tgfβ3^+/-^ CD group (left) are shown in red, and animals from the Tgfβ3^+/-^ TFD group (right) are shown in green. (C) Tgfβ3^+/-^ TFD vs. Tgfβ3^+/+^ TFD. Animals from the Tgfβ3^+/+^ TFD group (left) are shown in red, and animals from the Tgfβ3^+/-^ TFD group (right) are shown in green. The heatmap represents the intensity of significantly different compounds. All experiments were performed in whole kidneys of 4-month-old male mice (*n* = 7).

Gas chromatography-mass spectrometry (GC-MS) analysis identified 21 significantly upregulated metabolites in wild-type mice fed a TFD when compared with the CD group (Figure 8). These changes included increased levels of essential amino acids (isoleucine, phenylalanine, and threonine) and non-essential amino acids (alanine, cysteine, and proline). Among tricarboxylic acid (TCA) cycle-related metabolites, malic acid and fumaric acid were significantly elevated. Additionally, adenosine and oxalic acid showed significantly increased levels. Notably, glucose-6-phosphate, threonic acid, and urea were significantly altered exclusively in this comparison.

The TFD on mice with the deletion of Tgfβ3 showed 17 significant downregulated metabolites relative to Tgfβ3^+/−^ CD group (Figure 8). The results showed a significant reduction in essential amino acids (isoleucine, phenylalanine, and threonine) and non-essential amino acids (alanine, proline, and serine). Additionally, creatinine, hydroxyproline, gluconic acid, taurine, and malic acid followed the same trend, presenting a significant downregulation in the Tgfβ3^+/−^ TFD group versus the Tgfβ3^+/−^ CD group. In addition, betaine was uniquely identified as a significantly downregulated metabolite in this comparison.

Finally, the deletion of Tgfβ3 in mice fed the TFD revealed 19 significant downregulated compounds (Figure 8) compared to WT mice on the same diet. The downregulation was observed in essential amino acids (isoleucine, phenylalanine, and threonine) and non-essential amino acids (alanine, cysteine, aspartic acid, proline, and serine), as well as in hydroxyproline, gluconic acid, and taurine in the Tgfβ3^+/−^ TFD group relative to control mice on the same diet. In contrast, TCA cycle metabolites remained largely unchanged, although aspartic acid, maltotriose, and citric acid were significantly reduced.

P values and VIP values for each significant feature and comparison in LC-MS and GC-MS analyses are provided in Tables S8 and S9 (available as Excel files).

## Discussion

*Trans*-fats are mainly generated during the industrial hydrogenation of vegetable oils.^5^ Their consumption has been associated with increased risks of cancer or cardiovascular disease; consequently, the WHO encourages reducing their intake.^7^ Our research group has previously demonstrated that the partial absence of Tgfβ3 in mice kidneys leads to fibrosis, showing dysregulation of lipid metabolism and mitochondrial function.^18^

This work presents metabolomic, molecular, and cellular analyses that characterize the metabolic and lipid profiles in the renal tissue of wild-type mice fed a diet rich in *trans*-fats and the impact of lipid accumulation on fibrosis development induced by Tgfβ3 reduction. In this study, we found that TFD promoted renal damage through lipid accumulation without protective TG effects. This diet induced mitochondrial alteration, an increase of ROS species and oxidative stress due to the upregulation of malic acid and fumaric acid, TCA cycle metabolites. The presence of fibrosis due to the deletion of Tgfβ3 changed the process of lipid accumulation to large-sized lipid droplets with decreased long- and medium-chain TG. These changes exacerbated renal damage and were associated with reduced fatty acid oxidation, decreased taurine levels, and increased podocyte loss. This diet resulted in mitochondrial alterations, increased ROS production, and oxidative stress due to the upregulation of malic acid and fumaric acid, both TCA cycle metabolites, with a controlled 12-week intervention starting at 4 weeks of age. In other studies, using the same diet, significant metabolic changes have been reported with even shorter exposure periods,^20^^,^^21^ which supports the sufficiency of a 12-week exposure to the TFD to induce renal alterations.

High intake of SFAs from animal sources, common in western diets, has metabolic effects similar to those of trans fats.^22^ Industrial and ruminant *trans*-fats produce similar effects to this diet on human plasma lipoproteins, but only industrial *trans*-fat trigger inflammation and endoplasmic reticulum stress^6^ in preclinical models. Both fats increase total cholesterol and LDL, while decreasing HDL in clinical studies.^6^ In murine models, the impact of *trans* fats on lipoprotein and TG metabolism varies. Some studies showed increased total cholesterol, LDL, and TGs, with a decreased HDL,^6^^,^^23^ while others reported different outcomes. These variations highlight the importance of the type and quantity of trans fats and the choice of reference diets.^6^

The *trans*-fat-rich diet used in this study contains 76% mono- and polyunsaturated fatty acids (*cis* and *trans*), primarily derived from 274.1 g/kg of hydrogenated vegetable oil. Based on the manufacturer’s nutrient analysis, *trans*-fatty acids account for approximately 62 g/kg of diet, corresponding to ∼11–12% of total energy intake. According to WHO guidelines, *trans*-fat consumption should be limited to less than 1% of total daily energy intake (2.2 g/day in a 2000 kcal diet). Moreover, even a 2% increase in energy intake from *trans* fats has been associated with a 23% increase in cardiovascular disease incidence.^24^ Therefore, the proportion of industrial *trans* fats in the TFD markedly exceeds recommended intake thresholds and is sufficient to induce the metabolic and renal alterations observed in this study.

Although the pathophysiology of renal disease has previously been investigated in these studies, the impact of TFDs on the kidney lipidome of murine models has not been completely explored before. In our study, wild-type mice fed the *trans* diet showed decreased circulating and renal TGs, and most plasmalogens were upregulated. Paradoxically, these animals exhibited greater lipid accumulation of neutral lipids. These results contrast with the upregulation of TGs that Showalter et al. found when they analyzed the impact of an SFA diet on the kidney of a murine model without genetic alterations.^19^

**Figure 9:**
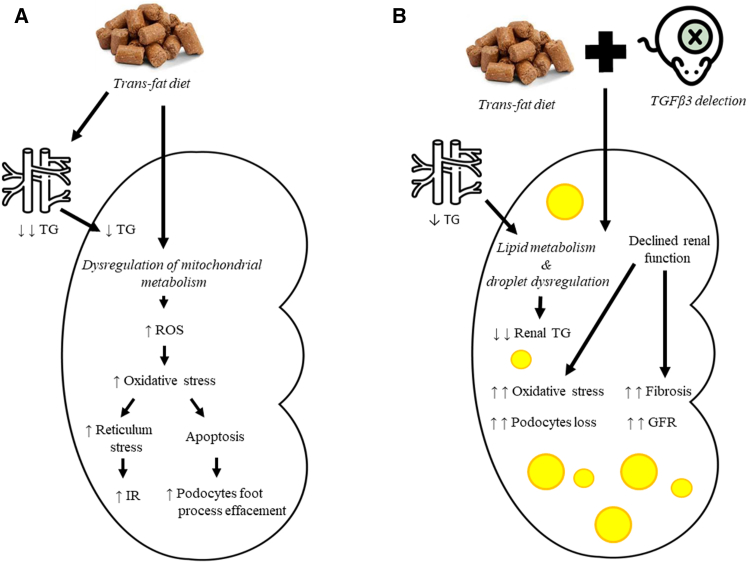
Schematic representation of renal alterations induced by *trans*-fat diet (A) and the aggravated damage resulting from Tgfβ3 deficiency under *trans*-fat dietary conditions (B) (A) Illustrates how *trans*-fat consumption impairs renal lipid metabolism, reduces triglyceride content, and increases oxidative stress and fibrosis. (B) Shows how the combination of *trans*-fat diet and Tgfβ3 heterozygosity leads to further lipid droplet dysregulation, podocyte loss, and exacerbated renal dysfunction. The figure has been adapted from ref.^6^^,^^22^ Abbreviations: ROS, reactive oxygen species; GFR, glomerular filtration rate; IR, insulin resistance.

TGs are known to play a protective role against lipotoxicity.^25^ Zambó et al. suggested that the intake of *trans* and saturated fats has similar metabolic effects, leading to greater toxicity due to a blockage in hepatic TG synthesis.^22^ In our study, TFD dysregulated mitochondrial metabolism with incipient oxidative stress, which likely triggered endoplasmic reticulum stress, contributing to insulin resistance and increased podocyte FPE (Figure 9).

In our results, the TFD drives a reduction in both serum and renal TG levels. Renal CD36 expression was not altered, and at the hepatic level, previous work from our group using the same dietary intervention^12^ showed increased hepatic expression of PPARγ and CIDEC genes in *trans*-fat-fed animals. This hepatic response is consistent with reduced TG export and, consequently, decreased availability of circulating TGs and reduced lipid supply to other organs. This effect may be further reinforced by the deleterious consequences of *trans*-fat intake, which has been proposed to impair TG synthesis at the hepatic level.^22^ Similar dissociations between circulating lipid levels and renal lipotoxicity have been described in CKD, which can progress irrespective of circulating lipid levels.^26^ However, we believe that in our model, TG depletion and renal accumulation of lipids in lipid droplets are due to impaired intracellular lipid handling caused by mitochondrial dysfunction, both through decreased beta-oxidation (decrease in PPARalpha and PGC1beta) and structural alterations (onion and doughnut appearance), induced by TGFβ3 deficiency and aggravated by a diet rich in *trans* fats.

In line with this concept, multi-omics studies indicate that renal injury is characterized by profound disturbances in lipid handling rather than by simple lipid overload.^27^^,^^28^ Lipotoxicity is known to profoundly affect renal lipid metabolism by increasing lipid synthesis while reducing fatty acid oxidation. When excess fatty acids are not oxidized, they are esterified with glycerol and become lipid droplets. This metabolic deregulation exacerbates the renal lipid deposition, leading to a state of energy depletion that finally causes cell apoptosis and contributes to CKD.^29^

It has been shown that patients with CKD present altered plasma amino acid profiles that dialysis cannot correct, and these alterations worsened as renal damage progresses.^30^ In our study, the *trans* diet increased polar metabolites in wild-type mice. Upregulation of polar metabolites could be a compensatory mechanism to retain amino acids due to high albuminuria present in the TFD, which is also supported by elevated GFR. Our research showed upregulated levels of isoleucine, proline, alanine and phenylalanine in mice fed TFD compared to their controls. Elevated serum levels of these amino acids are linked to insulin resistance and impaired renal function.^30^

In our study, the TFD induced a significant upregulation of fumaric and malic acid, along with a downregulation of mitochondrial gene expression in wild-type mice. Additionally, several exclusive lipids annotated in this comparison, including PG(39:1), PE(P-36:1), PE(36:3), and PE(O-36:4), are associated with mitochondrial metabolism. Perturbations in these lipids have been linked to impaired oxidative phosphorylation and mitochondrial dysfunction.^31^ These findings contrast with previous observations in liver and serum from mice fed animal fat diets.^32^ The concurrent increase in these lipid species, fumaric acid, malic acid, adenosine, and oxalic acid in TFD-fed mice suggests mitochondrial dysfunction, renal hyperfiltration, and fibrosis, consistent with metabolic alterations reported in patients with CKD with impaired TCA cycle function.^33^^,^^34^^,^^35^

Renal fibrosis is a complex pathological process that involves profound metabolic reprogramming. Growing evidence reinforces that renal fibrosis is not simply a structural alteration but also a metabolic and systemic disorder, in which metabolic impairment actively contributes and amplifies renal damage.^36^^,^^37^^,^^38^ In that context, TGFβ is a master regulator of renal fibrosis^39^ and multi-omics research has substantially expanded understanding of TGFβ/Smad-mediated renal injury by uncovering interactions with microbial ecosystems, metabolism, and other signaling cascades.^36^^,^^40^^,^^41^ The TGFβ superfamily includes three isoforms in mammals (TGFβ 1 to TGFβ3). In the human kidney, TGFβ2 and TGFβ3 are predominantly expressed in podocytes, while TGFβ1 is mainly expressed in the tubules.^42^ TGFβ1 is a profibrotic factor, responsible for the activation of fibroblasts to myofibroblasts, as well as EMT and macrophage-myofibroblast transition (MMT) processes, and the inhibition of metalloproteinases (MMPs) through PAI1, promoting the progression of kidney damage.^39^ However, the functions of TGFβ2 and TGFβ3 are not as clear. *In vitro* experiments have shown that all three isoforms are capable of inducing the synthesis of extracellular matrix components, and other studies have linked TGFβ2 to the regulation of both glucose and fatty acid metabolism.^43^ Furthermore, Cao and collaborators demonstrated that endogenous metabolites are capable of inducing EMT and MMT using untargeted metabolomics to identify metabolic signatures associated with renal fibrotic progression.^44^ Notably, canonical TGFβ/Smad signaling not only drives extracellular matrix deposition but also directly modulates metabolic pathways such as fatty acid oxidation, mitochondrial function, and lipid handling, thereby linking fibrotic signaling to metabolic rewiring.^36^^,^^39^ Recent multi-omics studies have demonstrated that alterations in lipid metabolism are not merely secondary events, but essential components of TGFβ-mediated renal injury.^38^^,^^41^ Therefore, TGFβ signaling emerges as a key element connecting lipid metabolic imbalance with the progression of renal fibrosis. In this context, previous studies of our laboratory with the model based on partial *Tgfβ3* deficiency showed the development of renal fibrosis, mitochondrial and lipid metabolic alterations through the activation of non-canonical pathways.^18^ In this study, we show that these alterations are further exacerbated when fibrosis-affected mice are subjected to an additional metabolic insult, such as a TFD, leading to more pronounced lipid dysregulation and podocyte loss.

The TFD in a fibrotic model exacerbated renal impairment in Tgfβ3^+/−^ mice compared to Tgfβ3^+/−^ CD, particularly impacting lipid metabolism (Figure 9). Although genes involved in lipid metabolism did not show significant changes in expression, lipidomic profiling revealed a significant decrease in TGs, protective lipids, and plasmalogens, compounds linked to oxidative stress and CKD in circulation.^45^ Notably, 23 distinct TG species were exclusively depleted in *trans*-fat-fed mice, a change that likely intensified lipid-induced oxidative stress^22^ and coincided with the elevated reactive oxygen species (ROS) levels observed in these animals.

The lipidomic analysis identified four lipid species with consistent alterations across experimental conditions, suggesting a potential role as descriptors of renal damage induced by dietary *trans*-fat exposure, TGFβ3 deficiency, or the combination of both factors. PS(P-40:4), which was decreased in all comparisons, has not been specifically highlighted in previous renal studies at the individual species level. However, plasmalogens have been proposed to exert protective roles against oxidative stress,^46^ and PS have been widely recognized as markers of cellular activation and apoptosis.^47^ PC (32:1), which was increased in our mice with renal damage, has been proposed as a predictor of hypertension^48^ and has been reported to be increased in the kidneys of ischemic rats^49^ as well as in rat models of adenine-induced CKD.^50^^,^^51^ On the other hand, PC (32:1) was found to be decreased in subjects following a low carbohydrate HFD^52^ highlighting it as a lipid highly sensitive to dietary composition and metabolic status. TG (51:1) and TG (53:1), which were consistently reduced in mice with renal damage, have been specifically associated with an increased risk of developing CKD in human cohort studies.^53^ Notably, TG (51:1) has also been found upregulated in patients with diabetic nephropathy,^54^ suggesting a complex regulation of TGs in relation to dietary intake and renal status. Altogether, the identification of these lipid species as a core signature of renal damage as indicators of altered redox balance, membrane remodeling, and TG handling in renal injury accentuates their translational relevance and justifies further targeted investigation in human cohorts.

The effect of Tgfβ3 deficiency in the obesogenic context worsens renal damage through different accumulation of lipid species and mechanisms. While Tgfβ1 and Tgfβ3 deficiencies are linked to similar processes^18^^,^^55^ and are exacerbated by high-fat diets in mice,^56^ Tgfβ3 deletion primarily impacts lipid metabolism gene expression. Notably, Tgfβ3^+/−^ TFD animals exhibited a significant increase in large lipid droplet accumulation relative to Tgfβ3^+/+^ TFD mice. Studies combining clinical, cellular, and animal models suggest that Tgfβ3 facilitates hyperplastic white adipose tissue expandability,^12^ a process in which adipocyte hypertrophy may result from the presence of large lipid droplets. In this context, Tgfβ3^+/−^ TFD mice, compared with Tgfβ3^+/−^ CD or Tgfβ3^+/+^ TFD animals, exhibited a marked imbalance in lipid metabolism, increased oxidative stress, higher OPA1 expression, and a greater number of enlarged lipid droplets. It has been proposed that mitochondrial oxidative damage reprograms renal lipid metabolism, promoting lipid droplet formation,^57^ a mechanism that is consistent with our observations.

*In vitro* studies performed in podocytes and proximal tubular epithelial cells (MCTs) with siRNA-mediated silencing of Tgfβ3 have shown increased lipid accumulation together with the reduced expression of PPARα, a key regulator of mitochondrial fatty acid β-oxidation.^18^ In addition, unpublished data from the same cellular models revealed a significant reduction in TFAM expression, recently proposed as a critical guardian of mitochondrial integrity and cellular homeostasis.^58^ These findings support the concept that TGFβ3 deficiency leads to mitochondrial dysfunction and impaired lipid oxidation, favoring intracellular lipid accumulation. Under obesogenic conditions, such as exposure to a TFD, these alterations may contribute to the formation and persistence of enlarged lipid droplets observed in Tgfβ3^+/−^ mice. It has been proposed that mitochondrial oxidative damage reprograms renal lipid metabolism, promoting lipid droplet formation,^57^ a mechanism that is consistent with our observations. Therefore, preferential accumulation of lipids within enlarged lipid droplets may play a crucial role in the progression of renal damage in the presence of preexisting fibrosis. These findings underscore the pivotal role of large lipid droplet accumulation in the progression of renal pathology, beyond general lipid accumulation, as highlighted in other studies.^59^

Tgfβ3^+/−^ TFD vs. Tgfβ3^+/−^ CD and Tgfβ3^+/−^ TFD vs. Tgfβ3^+/+^ TFD comparisons revealed several overlapping features indicative of exacerbated renal injury. The increased ROS production observed in these animals could explain the significant podocyte loss obtained between these groups, also described in mice fed an SFA diet,^60^ a process associated with progressive nephropathy in lethal podocyte injury.^61^ At the mitochondrial level, no major differences were observed in the expression of mitochondrial genes in these comparisons. However, alterations in TCA cycle metabolites were detected, including a reduction in malic acid in Tgfβ3^+/−^ TFD compared with Tgfβ3^+/−^ CD mice, suggesting functional metabolic alterations rather than transcriptional regulation. It is important to note that aberrant mitochondrial shapes, associated with an altered electron chain,^62^^,^^63^ were found in Tgfβ3^+/−^ CD, Tgfβ3^+/−^ TFD, and Tgfβ3^+/+^ TFD mice, possibly related to the increased ROS accumulation observed in these animals.

Adenosine and oxalic acid, polar metabolites previously associated with renal damage when elevated,^34^^,^^35^ did not follow this expected pattern between these groups. Despite a significant reduction in adenosine levels in one comparison, this change does not align with its described pathogenic role, which is linked to elevated concentrations. Therefore, these metabolites may not serve as reliable markers of renal impairment in this specific context. However, Tgfβ3^+/−^ TFD vs. Tgfβ3^+/+^ TFD and Tgfβ3^+/−^ TFD vs. Tgfβ3^+/−^ CD comparisons revealed a decrease in taurine, a key metabolite that provides renal protection against proteinuria^64^ and reduces ROS levels in glomerular disease models.^65^ In general, patients with kidney damage have decreased levels of taurine.^66^ Our results suggest that Tgfβ3 deficiency reduces renal availability of taurine, potentially worsening the condition even without the effect of TFD.

CKD represents a major global health burden, affecting approximately 10% of the worldwide population,^67^ highlighting the importance of establishing translational bridges between experimental models and human cohorts. In line with the translational relevance suggested by the core lipid signature discussed above, human studies from our lab comparing individuals with severe obesity and CKD versus obese subjects without renal impairment have reported increased levels of TGs, diacylglycerols, PCs, and essential amino acids, proposing a specific PC(35:3) as a potential descriptor of renal damage that do not normalize after bariatric surgery.^68^ Dysregulation of PC metabolism has been demonstrated to be involved in CKD pathology.^51^ Together, these findings, along with our identification of PC (32:1), highlight PCs as lipid species of particular interest that warrant further detailed investigation as part of potential biomarker panels for CKD. Similarly, patients with Alport syndrome, a rare inherited genetic disorder with progressive loss of kidney function, display a generalized increase in circulating lipid species compared with healthy controls.^54^ Although CKD is commonly associated with elevated circulating TGs,^69^ it is noteworthy that subjects following a low-carbohydrate, high-fat diet exhibit a reduction in serum TGs attributed to metabolic reprogramming characterized by reduced *de novo* lipogenesis and increased fatty acid oxidation.^52^ This observation parallels our findings, in which increased lipid intake does not translate into higher TG levels but rather into altered lipid handling. In addition, taurine depletion, observed in TGFβ3-deficient mice under *trans*-fat dietary conditions, has been also reported in human CKD.^70^ However, other human studies proposing lipid and metabolite markers for different forms of renal damage do not consistently identify the lipid species comprising our core signature.^71^^,^^72^ This lack of concordance, even among studies addressing similar renal pathologies, underscores the need for standardized analytical approaches, as even metabolite trends may differ depending on sample type (plasma versus serum) in the same subjects.^73^

Although C57BL/6 mice are relatively resistant to kidney damage,^74^ this strain is widely used to evaluate renal injury in various experimental models.^75^^,^^76^ The heterozygous Tgfβ3 mouse develops clear signs of renal dysfunction, as previously reported.^18^ Therefore, our aim was to assess a sensitized model in which the impact of *trans*-fat exposure on renal health could be evaluated. This genotype mimics human susceptibility to diet-induced renal alterations.

In conclusion, the use of specific diets in animal models, whether in wild-type or different genetically modified contexts, demands detailed analyses capable of characterizing metabolic and lipid profiles comprehensively. Such approaches enable a deeper understanding and phenotyping of the metabolic effects and the underlying mechanisms driven by diets of varying compositions, as well as the introduced genetic modifications. This study demonstrates that, in the kidney under obesogenic conditions, the reduction of TGFβ3 expression contributes to tissue dysfunction. Specifically, it leads to mitochondrial failure associated with defects in lipid droplet formation and changes in the patterns of lipid types accumulated in lipid droplets. Notably, the worsening of renal function appears to be directly linked to the increased accumulation of large lipid droplets, a factor that warrants further investigation.

### Limitations of the study

Several limitations of this study should be acknowledged. First, the most relevant lipid species highlighted in this work were annotated using an untargeted approach. These findings would need to be validated using authentic reference standards to strengthen the proposed core lipid signature of renal damage. However, it is important to note that such standards are not commercially available for all lipid species. Second, although *in vitro* studies using renal cells have demonstrated that genes involved in fatty acid β-oxidation and mitochondrial integrity are altered upon Tgfβ3 deficiency, the precise mechanisms driving the formation of enlarged lipid droplets remain unresolved and should be addressed in future studies. Third, all experiments were performed in male mice. The potential influence of sex on the observed phenotypes was not evaluated and represents a limitation of this study. Finally, the mechanistic basis underlying the TG depletion observed in response to the TFD remains incompletely understood. Although our data and previous studies suggest alterations in lipid handling and TG metabolism, the precise pathways responsible for this phenomenon warrant further investigation.

## Resource availability

### Lead contact

Requests for further information and resources should be directed to and will be fulfilled by the lead contact, Gema Medina-Gomez (gema.medina@urjc.es).

### Materials availability

This study did not generate new unique reagents or materials. The global homozygous Tgfβ3 mouse model was previously generated and has been described elsewhere. Details of the primers used for RT-qPCR are provided in the Supplemental Information. Therefore, all reagents and resources used in this study are commercially available or have been previously described.

### Data and code availability

• Metabolomics data are available at Metabolomics Workbench (Metabolomics Workbench: ST003738) and are publicly available as of the date of publication.
• This study used custom analysis scripts for data processing and statistical analyses, including MATLAB-based pipelines routinely applied at the Metabolomics and Bioanalysis Center (CEMBIO) and a custom Python-based image analysis workflow for lipid droplet quantification. These scripts are available from the lead contact upon reasonable request.
• Any additional information required to reanalyze the data reported in this paper is available from the lead contact upon request.

## Acknowledgments

The research presented in this work was supported by Ministerio de Economía y Competitividad de España [ PID2020-116875RB-I00, PDC2021-121871-I00, RED2022-134313-T], 10.13039/100012818Comunidad de Madrid (Spain), [S2017/BMD-3684 MOIR2-CM and S2022/BMD-7227 MOIR-ACTOME-CM]. We thank Emilio Conde for his valuable contribution to the development of the image analysis script used for the quantification and sizing of lipid droplets.

## Author contributions

B.L., E.E., A.I.L., A.G., and G.M.-G. designed the study; E.E. and P.C. performed animal monitoring and care; E.E., A.I.L., and P.C. collected the samples; B.L., E.E., and A.I.L. performed the experiments; B.L., E.E., C.G.R., F.J.R., and D.H. analyzed the data; B.L. and A.I.L. prepared the figures; B.L., F.J.R., G.M.-G., A.I.L., and A.G. drafted and revised the paper. All authors approved the final version of the manuscript.

## Declaration of interests

The authors declare no competing interests.

## Declaration of generative AI and AI-assisted technologies in the writing process

During the preparation of this work, the authors used ChatGPT to assist with language editing and to improve the clarity and fluency of the text. After using this tool, the authors reviewed and edited the content as needed and take full responsibility for the content of the published article.

## STAR★Methods

### Key resources table

**Table:** 

REAGENT or RESOURCE	SOURCE	IDENTIFIER
**Antibodies**		

α-SMA	Dako	M0851 ; RRID: AB_2223500
Anti-Mouse	Bio-Rad	170-6516; RRID: AB_11125547
Anti-Rabbit	Bio-Rad	170-6515; RRID: AB_11125142
CD36	Santa Cruz	Sc7309; RRID: AB_627044
Nitrotirosine	Milipore	06-284; RRID: AB_310089

**Experimental Models: Cell lines**		

Mouse immortalized podocyte cell line	Izquierdo-Lahuerta A. and Coward R. laboratories	Not commercially available

**Experimental Models: Organism/Strains**		

Mouse C57BL/6J	Martinez laboratory (Universidad Complutense de Madrid)	https://doi.org/10.1111/j.1432-0436.2007.00226.x.
Mouse Tgfβ3^+/−^ (global heterozygous)	Martinez laboratory (Universidad Complutense de Madrid)	https://doi.org/10.1111/j.1432-0436.2007.00226.x.

**Chemicals, peptides, and recombinant proteins**		

Control diet (10% kcal fat)	Research Diets	D12450B
*Trans*-fat diet (29% fat; 54.4% kcal)	Envigo	TD.07011
Palmitic acid	Sigma-Aldrich	P0500
D-Glucose	Sigma-Aldrich	G7021
Dihydroethidium (2-HE)	Sigma-Aldrich	37291
BODIPY neutral lipid probe	Thermo Fisher Scientific	D3922
Collagenase (Clostridium histolyticum)	Sigma-Aldrich	C5138
Human insulin	Novo Nordisk	Actrapid

**Deposited data**		

Metabolomic and lipidomic datasets	Metabolomics Workbench	ST003738

**Software and Algorithms**		

Agilent MassHunter Acquisition	Agilent Technologies	B.08.00
Agilent MassHunter Profinder	Agilent Technologies	B.08.00
MassHunter Unknown Analysis	Agilent Technologies	B.07.00
MassProfiler Professional	Agilent Technologies	B.12.1
GraphPad Prism	GraphPad Software	Version 8
SIMCA-P	Umetrics	Version 15.0
Metaboanalyst	Xia Lab	Version 5.0
ImageJ	National Institutes of Health	Version 1.45
MATLAB	MathWorks	Custom MATLAB scripts, available upon request
Python	Python Software Foundation	Custom Python scripts, available upon request
LION	Lipid Ontology	https://www.lipidontology.com/
Venny	CNB-CSIC	Version 2.1

**Other**		

UHPLC-QTOF-MS platform	Agilent Technologies	Infinity 1290 + 6545 Q-TOF
GC-QTOF-MS platform	Agilent Technologies	7890B GC + 7250 Q-TOF

### Experimental model and study participant details

#### Cell lines and treatments

An immortalized mouse podocyte cell line was used for *in vitro* experiments. This cell line was established by Dr. Adriana Izquierdo Lahuerta (Universidad Rey Juan Carlos, Madrid, Spain) in collaboration with Dr. Richard Coward's laboratory (University of Bristol, Bristol, UK). These cells proliferate at 33°C and transition to a quiescent and differentiated phenotype when cultured at 37°C for 10–14 days. The cell line was maintained in RPMI medium (Sigma-Aldrich, St. Louis, MO, USA) supplemented with 10% fetal bovine serum (FBS; Gibco, Thermo Fisher Scientific, Waltham, MA, USA) and penicillin (100 U/mL)/streptomycin (100 μg/mL) (Gibco, Thermo Fisher Scientific, Waltham, MA, USA).

Cells were authenticated using podocin and nephrin as specific podocyte markers by immunofluorescence and RT-PCR. Mycoplasma contamination is routinely assessed by PCR using specific Mycoplasma primers on culture supernatants.

#### Animal models

Wild-type (Tgfβ3^+/+^) C57BL/6J mice and heterozygous global murine model of Tgfβ3 (Tgfβ3^+/−^) were used in this study. All experiments were performed in male mice. Wild-type mice were obtained at 4 weeks of age and fed either a control diet (CD) with 10% fat (D12450B, Research Diets, New Brunswick, NJ, USA) or a high *trans*-fat diet (TFD) containing 29% fat and providing 54.4% of total kcal (TD.07011, Envigo, Indianapolis, IN, USA) was administered from 4 to 16 weeks of age. The fatty acid profile of the TFD comprised 24% saturated, 61% monounsaturated (cis + trans), and 15% polyunsaturated (cis + trans) fatty acids, derived primarily from 274.1 g/kg of hydrogenated vegetable oil. Based on the manufacturer’s extended nutrient analysis, *trans*-fatty acids constitute approximately 50% of the MUFA fraction and 30% of the PUFA fraction of the diet, corresponding to an estimated 62 g/kg of diet (∼11–12% of total energy intake). This confirms that trans fats represent a substantial proportion of the lipid fraction and clearly exceed recommended intake thresholds.

*Tgfβ3*^+/−^ mice, previously characterized and associated with renal damage,^18^ was also studied under the same dietary conditions (CD or TFD) from 4 to 16 weeks of age. Complete *Tgfβ3* deletion (knockout) is lethal within days after birth. At 16 weeks, kidneys were dissected, weighed, and frozen in liquid nitrogen for further analyses.

All animal procedures were approved by the Research Ethics Committee of Universidad Rey Juan Carlos and complied with national and institutional guidelines. Experimental procedures were also authorized by the Comunidad de Madrid (PROEX 194/17 and PROEX 041.7/23).

#### Ethical statement

All animal protocols used in this study were approved by the Research Ethics Committee of the Universidad Rey Juan Carlos and complied with relevant animal welfare laws, guidelines and policies. Experimental procedures were also authorized by the Comunidad de Madrid under the registration numbers 0912202000421 (PROEX 194/17) and 1701202304423 (PROEX 041.7/23).

### Method details

#### Cell treatments

Prior to experiments, podocytes were deprived of serum for 12 h. Then, cells were incubated for 24 h either with vehicle medium (Vh) or under different caloric stimuli: 500 μM palmitic acid (PA; Sigma-Aldrich, St. Louis, MO, USA) and/or 25 mM high glucose (HG; Sigma-Aldrich, St. Louis, MO, USA), as previously described.^77^

#### Metabolic and renal functional analyses

Basal glucose levels were measured using an AlphaTRAK glucometer and strips (Zoetis, Parsippany, NJ, USA), and urine was collected over 24 h using Tecniplast metabolic cages (Tecniplast, Buguggiate, Italy). Metabolic parameters were determined at IdiSNA (Pamplona, Spain), and glomerular filtration rate (GFR) was assessed by iohexol plasma clearance at the University of La Laguna (San Cristobal de la Laguna, Tenerife, Spain). For insulin sensitivity tests, mice underwent a 14-h fast and received intraperitoneal injections of 10 U/kg human insulin (Actrapid; Novo Nordisk, Bagsværd, Denmark) or saline.

#### Gene expression analysis

Total RNA was extracted from kidneys following Martínez-García et al.,^78^ and gene expression was quantified by RT-PCR using β-actin, 36B4, 18S, and B2M as housekeeping genes, which were validated with BestKeeper software (BestKeeper software, Pfaffl Lab).^79^

#### Histology, immunohistochemistry, flow cytometry and microscopy

For histological evaluation, Picrosirius Red staining (Sigma-Aldrich, St. Louis, MO, USA) was used to distinguish collagen fibers, and Oil Red O staining (Sigma-Aldrich, St. Louis, MO, USA) was used to detect neutral lipids in cryosections.

Immunohistochemistry was performed on paraffin sections using α-SMA (Dako, Glostrup, Denmark) and Nitrotyrosine antibodies (Millipore, Burlington, MA, USA) and quantified with ImageJ (version 1.45; National Institutes of Health, Bethesda, MD, USA).

Flow cytometry was employed to measure superoxide anion (dihydroethidium; Sigma-Aldrich, St. Louis, MO, USA), intracellular lipids (BODIPY 493/503; Thermo Fisher Scientific, Waltham, MA, USA), lipid transport (anti-CD36; Santa Cruz Biotechnology, Dallas, TX, USA), and podocyte counts (anti-podocin; Abcam, Cambridge, UK) in digested kidney samples on a Beckman Coulter Cytomics FC500 MPL (Beckman Coulter, Brea, CA, USA).

Transmission electron microscopy (TEM) was conducted on ultra-thin kidney sections to assess glomerular basement membrane (GBM) thickness and foot process effacement (FPE) using ImageJ software (version 1.45; National Institutes of Health, Bethesda, MD, USA).

#### Metabolomic and lipidomic analyses

Untargeted metabolomic and lipidomic analyses were performed in whole kidney samples using liquid chromatography-mass spectrometry (LC-MS) and gas chromatography-mass spectrometry (GC-MS) as previously reported by Cala et al. and Medina-Gómez et al.^80^^,^^81^ LC-MS was conducted in both positive and negative ion modes using a UHPLC-QTOF-MS system (Agilent Technologies, Santa Clara, CA, USA), while GC-MS was performed in an EI-QTOF-MS system (Agilent Technologies, Santa Clara, CA, USA). Quality control (QC) samples were injected throughout the analytical measurements to monitor stability.

#### Feature annotation in omics analyses

Untargeted omics analyses allowed the annotation of a total of 76 metabolites by GC-MS and 177 lipid species by LC-MS.

For GC-MS, annotation was performed as part of the signal-processing workflow (Figure S4). Total ion chromatograms (TICs) were first inspected to assess chromatographic quality and the signal of the internal standard. Data files were then formatted for MassHunter Quantitative using MassHunter Workstation GC/MS software (B.04.01, Agilent Technologies, Santa Clara, CA, USA), and deconvolution and compound annotation were carried out using MassHunter Unknown Analysis (B.7.00, Agilent Technologies, Santa Clara, CA, USA). Chemical assignment was achieved by comparing deconvoluted mass spectra and measured retention indices against the Fiehn libraries (version 2008; West Coast Metabolomics Center, University of California, Davis, CA, USA) and a custom in-house library generated at CEMBIO. This annotation was complemented using the commercial NIST spectral library (version 2.2; National Institute of Standards and Technology, Gaithersburg, MD, USA). Identities with high spectral matching scores (>80%) and concordant retention indices on the n-alkane scale were putatively annotated.

For LC-MS, only statistically significant features identified by univariate and multivariate analyses were annotated. Annotation was performed by combining information from spectral databases with MS/MS fragmentation data acquired at 20 and 40 eV in both positive and negative ionization modes. Fragment ions were evaluated using multiple databases, including HMDB, MassBank, MetFrag, PubChem, and additional bibliographic resources. Furthermore, diacylglycerols (DG) and triacylglycerols (TG) were annotated in a putative manner by evaluating the different adducts formed by these lipid species. The use of LC-MS in negative ionization mode also allowed verification of acyl chain length for these lipids.

### Quantification and statistical analysis

Biochemical, gene expression, and tissue analyses: Normality was assessed with the Shapiro-Wilk test. For normally distributed data, parametric two-way ANOVA was used; otherwise, a non-parametric test (Kruskal-Wallis) was applied. Pairwise comparisons were adjusted using the Benjamini-Hochberg FDR procedure. Metabolomic analyses: Multivariate analysis (MVA) was performed in SIMCA-P (v15.0), and univariate analysis (UVA) with a custom MATLAB script. The value of n represents the number of biological replicates or animals analyzed per experimental group, as indicated in the figure legends or table footnotes.

Detailed metabolomics procedures, including LC-MS and GC-MS analyses, are provided in the Supplementary Methods. The supplementary materials further include a Venn diagram, summary tables of LC-MS and GC-MS analytical conditions, dedicated tables summarizing sample preparation and signal processing workflows for both techniques, lipid metabolic pathway representations, primer and antibody tables, and lists of significant metabolites (available as Excel files).

## Supplemental information

Document S1. Figures S1–S7; Methods S1; Tables S1–S7

Table S8. Statistically significant metabolites in kidney samples from comparisons of Tgfβ3^+/+^ TFD vs. Tgfβ3^+/+^ CD and Tgfβ3^+/−^ CD vs. Tgfβ3^+/+^ CD analyzed by LC-MS and GC-MS

Table S9. Statistically significant metabolites in kidney samples from comparisons of Tgfβ3^+/−^ TFD vs. Tgfβ3^+/−^ CD and Tgfβ3^+/−^ TFD vs. Tgfβ3^+/+^ TFD analyzed by LC-MS and GC-MS

## Footnotes

Supplementary data related to this article can be found online at https://doi.org/10.1016/j.isci.2026.116506.
