# Supplementary material for: Trans fatty acids alter renal health in TGFβ3 mice with fibrosis revealed by metabolomics and lipidomics
Source: iScience. 2026 Jun 30;29(7):116506. doi: 10.1016/j.isci.2026.116506 (PMC13378014; doi:10.1016/j.isci.2026.116506)
Supplement: Document S1. Figures S1–S7; Methods S1; Tables S1–S7 [file mmc1.pdf]

iScience, Volume *29*

Supplemental information

Trans fatty acids alter renal health in TGFβ3 mice with fibrosis revealed by metabolomics and lipidomics

Borja Lanzon, Elia Escasany, Almudena G. Carrasco, Carolina Gonzalez-Riano, Daniel Horrillo, Patricia Corrales, Antonia García, Francisco J. Ruperez, Adriana Izquierdo-Lahuerta, and Gema Medina-Gomez

Supplemental Information

Supplemental Figures

Figure S1: Venn diagram of differentially abundant lipid species across genotype and diet comparisons.

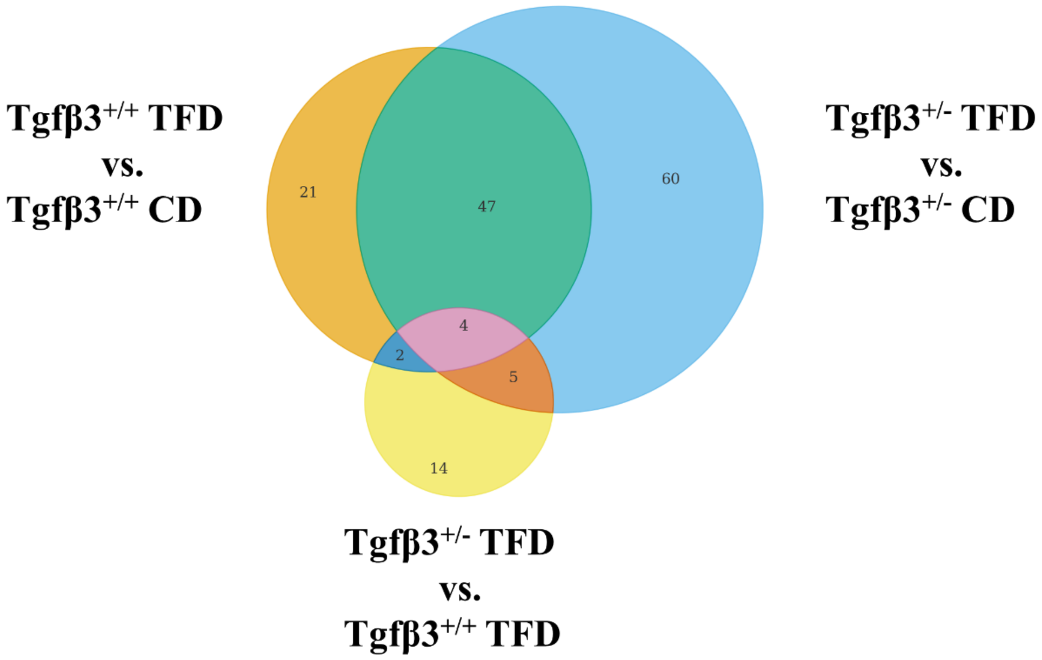

Venn diagram showing the distribution of differentially abundant lipid species across the three comparisons: Tgfb3+/+ TFD vs Tgfb3+/+ CD (orange), Tgfb3+/− TFD vs Tgfb3+/− CD (blue), and Tgfb3+/− TFD vs Tgfb3+/+ TFD (yellow). Four lipid species were commonly altered in all three conditions, while others were unique to individual comparisons or shared between two conditions, reflecting both shared and condition-specific lipidomic alterations. Values indicate the number of lipids within each intersection.

Figure S2: Lipid metabolic pathway alterations in Tgfβ3$^{+/+}$ mice under trans-fat diet conditions. Metabolic pathway of the significant lipids identified in the comparison between Tgfβ3$^{+/+}$ TFD and Tgfβ3$^{+/+}$ CD groups.

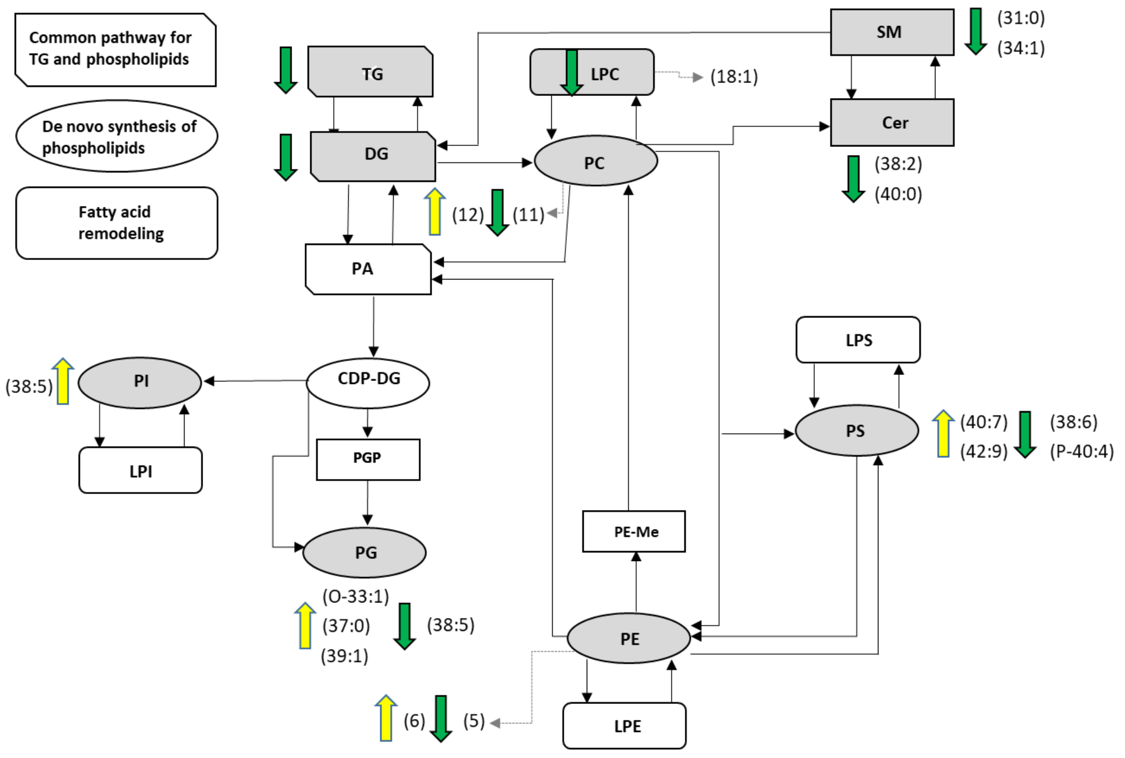

Color coding (yellow and green) indicates increased or decreased lipid abundance, respectively. P-values and VIP values for each lipid species are reported in Table S3. Abbreviations: TG, triglycerides; DG, diglycerides; PC, phosphatidylcholine; LPC, lysophosphatidylcholine; Cer, ceramide; SM, sphingomyelin; PA, phosphatidic acid; PI, phosphatidylinositol; LPI, lysophosphatidylinositol; PE, phosphatidylethanolamine; LPE, lysophosphatidylethanolamine; PS, phosphatidylserine; LPS, lysophosphatidylserine; PE-Me, N-methyl phosphatidylethanolamine.

Figure S3: Lipid metabolic pathway alterations in Tgfb3$^{+/-}$ mice under trans-fat diet conditions. Metabolic pathway of the significant lipids identified in the comparison between Tgfb3$^{+/-}$ TFD and Tgfb3$^{+/-}$ CD groups.

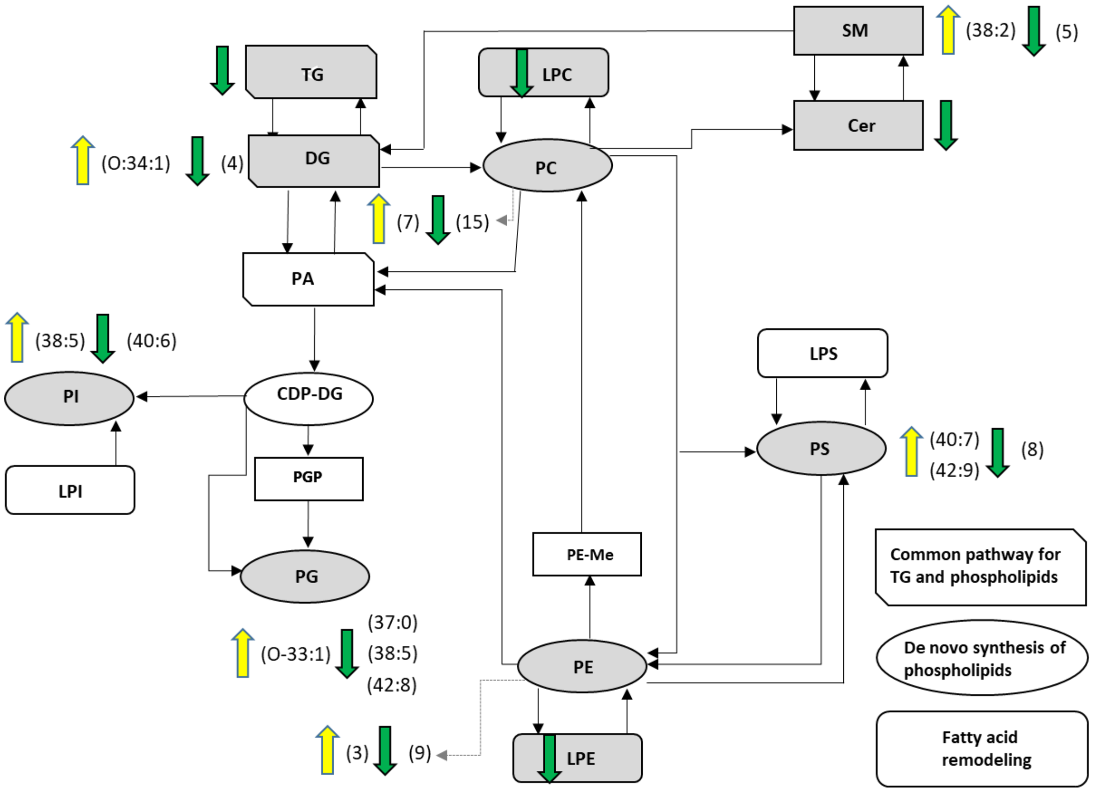

Color coding (yellow and green) indicates increased or decreased lipid abundance, respectively. P-values and VIP values for each lipid species are reported in Table S4. Abbreviations: TG, triglycerides; DG, diglycerides; PC, phosphatidylcholine; LPC, lysophosphatidylcholine; Cer, ceramide; SM, sphingomyelin; PA, phosphatidic acid; PI, phosphatidylinositol; LPI, lysophosphatidylinositol; PE, phosphatidylethanolamine; LPE, lysophosphatidylethanolamine; PS, phosphatidylserine; LPS, lysophosphatidylserine; PE-Me, N-methyl phosphatidylethanolamine.

Figure S4: Lipid metabolic pathway alterations associated with Tgfb3 deficiency under trans-fat diet conditions. Metabolic pathway of the significant lipids identified in the comparison between Tgfb3$^{+/-}$ TFD and Tgfb3$^{+/+}$ TFD groups.

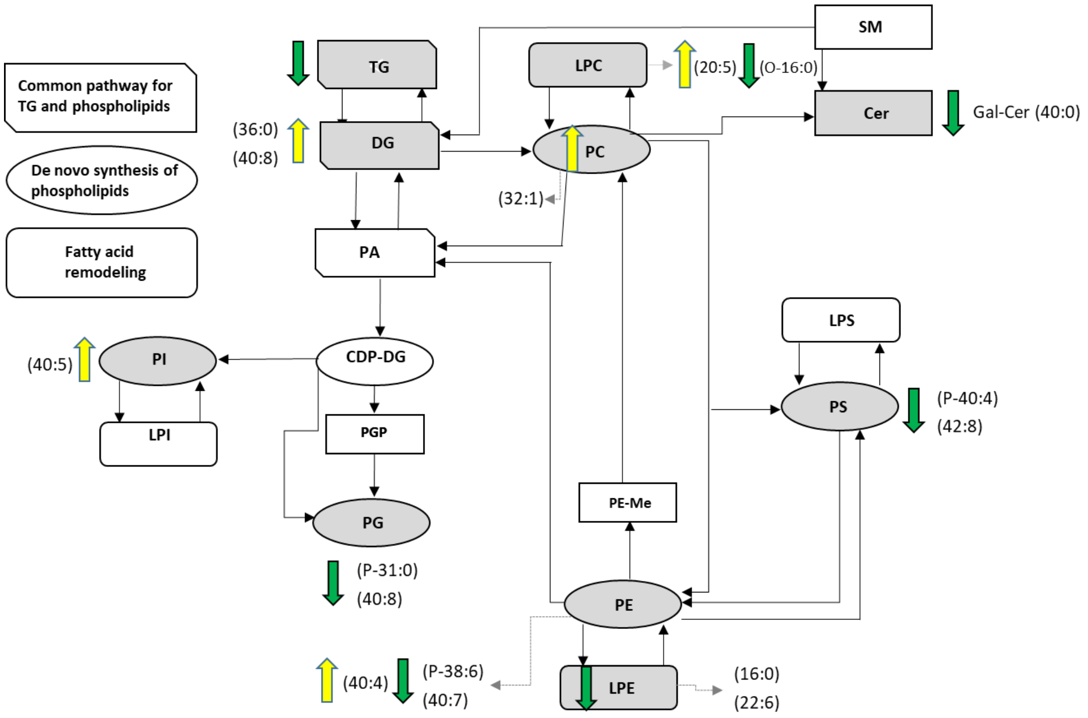

Color coding (yellow and green) indicates increased or decreased lipid abundance, respectively. P-values and VIP values for each lipid species are reported in Table S4. Abbreviations: TG, triglycerides; DG, diglycerides; PC, phosphatidylcholine; LPC, lysophosphatidylcholine; Cer, ceramide; SM, sphingomyelin; PA, phosphatidic acid; PI, phosphatidylinositol; LPI, lysophosphatidylinositol; PE, phosphatidylethanolamine; LPE, lysophosphatidylethanolamine; PS, phosphatidylserine; LPS, lysophosphatidylserine; PE-Me, N-methyl phosphatidylethanolamine.

Supplemental Methods

Methods S1: Metabolomics analysis procedures.

Untargeted metabolomic analyses were performed using LC-MS and GC-MS platforms as previously described. Kidney samples were processed following established protocols for tissue metabolomics, including homogenization, extraction, and phase separation.

For LC-MS analysis, samples were analyzed using an UHPLC system coupled to a Q-TOF mass spectrometer operating in both positive and negative electrospray ionization modes. For GC-MS analysis, samples were derivatized and analyzed using a gas chromatography system coupled to a Q-TOF mass spectrometer operating in electron impact mode.

Detailed analytical conditions for LC-MS and GC-MS, including chromatographic separation, ionization parameters, and mass spectrometry settings, are summarized in **Tables S1, S2, S3, S4 and S5**. Data processing workflows are illustrated in **Figures S5, S6** and **S7**.

Associated methodological workflows

Figure S5: Workflow for kidney sample preparation for LC-MS and GC-MS metabolomic analyses. Schematic overview of tissue processing, extraction, and sample preparation steps prior to chromatographic analysis.

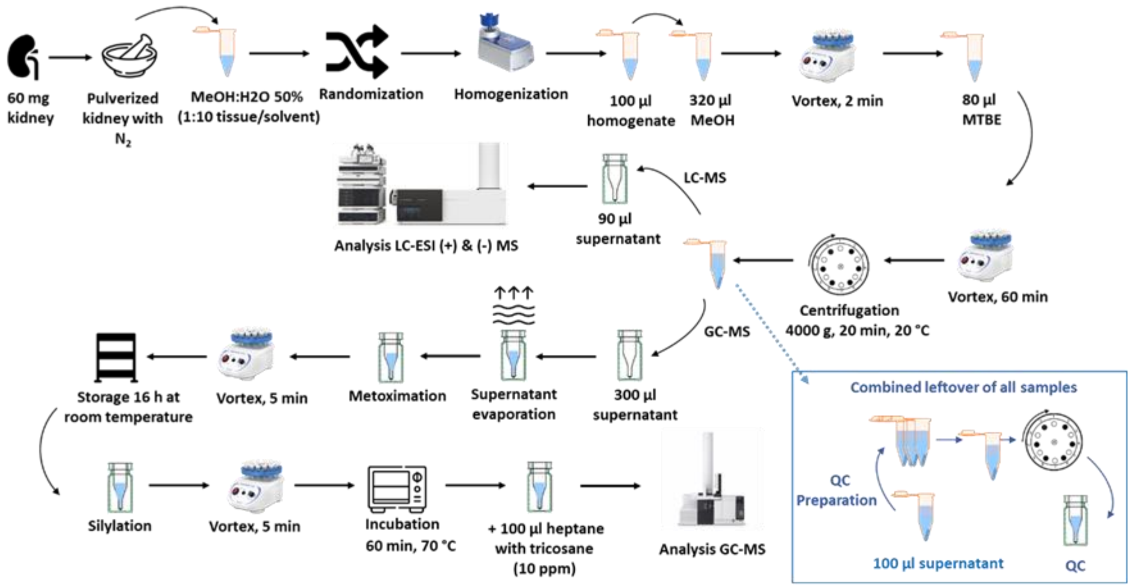

Figure S6: Workflow for signal processing in LC-MS analysis. Schematic overview of feature extraction, filtering, and data processing steps applied to LC-MS datasets.

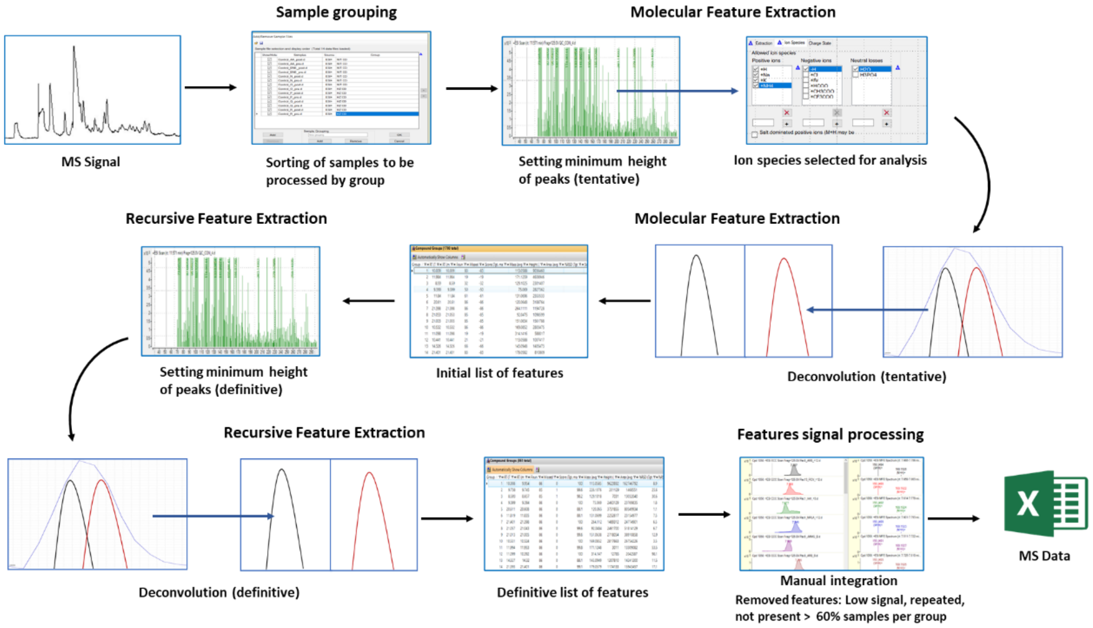

Figure S7: Workflow for signal processing in GC-MS analysis. Schematic overview of deconvolution, feature annotation, and data processing steps applied to GC-MS datasets.

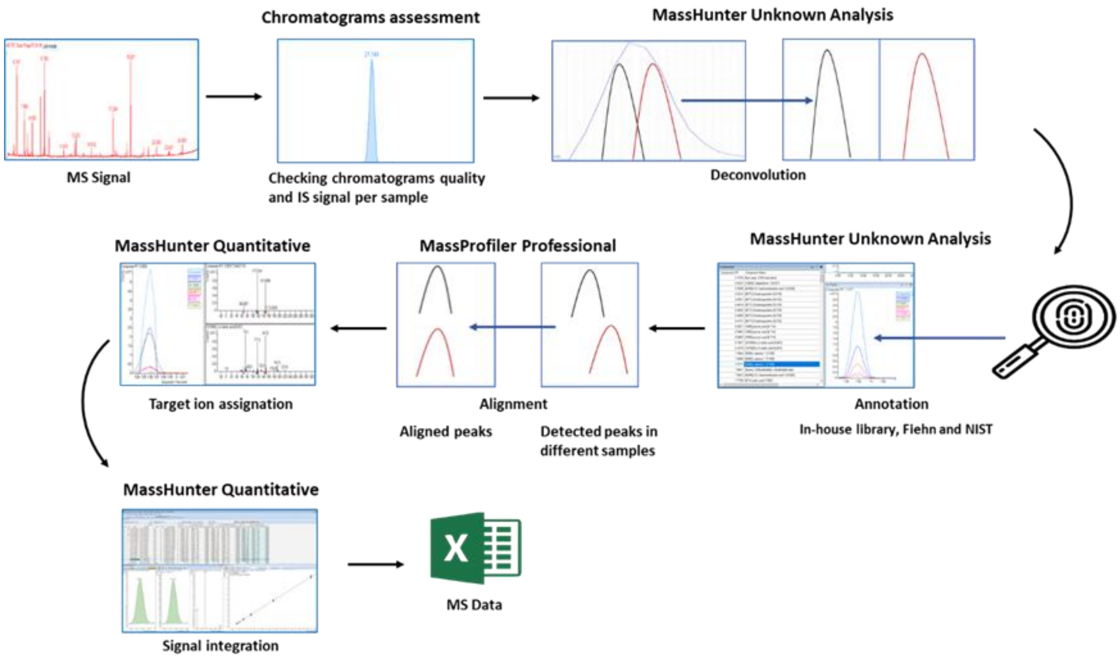

Enrichment and pathway analyses were performed using LION/web and MetaboAnalyst 5.0. Venn diagrams were generated using Venny 2.1. Lipid metabolic pathways were adapted from WikiPathways.

Supplemental Tables

Table S1: Main LC-MS analytical conditions used for lipidomic analysis.

Parameter	Condition
Instrument	Agilent 1290 UHPLC coupled to 6545 Q-TOF MS
Ionization	ESI (+) and ESI (−)
Column	Poroshell 120 Infinity Lab EC-C8 (2.1 × 150 mm, 2.7 µm)
Precolumn	Supelco Ascentis Express C8 (0.5 cm × 2.1 mm, 2.7 µm)
Column temperature	60 °C
Flow rate	0.5 mL/min
Injection volume	1 µL (+), 1.5 µL (−)
Autosampler temperature	15 °C
Mobile phase A (+)	H2O + 10 mM NH_4HCO_2
Mobile phase B (+)	MeOH:IPA (85:15) + 10 mM NH_4HCO_2
Mobile phase A (−)	H2O + 0.1% formic acid
Mobile phase B (−)	MeOH:IPA (85:15) + 0.1% formic acid
Gradient	75% to 96% B (0–31 min), 100% B (31.5–32.5 min), re-equilibration to 40 min
Mass range	100–1700 m/z
Capillary voltage	3500 V
Nozzle voltage	1500 V
Gas temperature	370 °C
Gas flow	11 L/min

Table S2: Main GC-MS analytical conditions used for metabolomic analysis.

Parameter	Condition
Instrument	Agilent 7890B GC coupled to 7250 Q-TOF MS
Injector	Gerstel MPS automatic injector
Injection mode	Split (1:12)
Injection volume	1 µL
Column	30 m × 0.25 mm × 0.25 µm (95% dimethyl–5% diphenyl polysiloxane)
Carrier gas	Helium
Flow rate	0.938 mL/min
Oven program	60 °C (1 min), ramp to 325 °C at 10 °C/min, hold 10 min
Injector temperature	250 °C
Ionization	Electron impact (70 eV)
Filament temperature	200 °C
Mass range	40–600 m/z

Table S3: Sample preparation for LC-MS and GC-MS analysis.

Step	LC-MS	GC-MS
Sample type	Kidney tissue (~60 mg)	Kidney tissue (~60 mg)
Initial extraction	50% $MeOH:H_2O$ (1 mg:10 µL), homogenization (Qiagen TissueLyser LT)	Same extraction as LC-MS
Protein precipitation	Addition of methanol (vortex 2 min)	Same
Lipid extraction	MTBE addition, 1 h mixing, centrifugation (4000 g, 20 min, 20°C)	Same
Phase used	Supernatant directly used	Supernatant collected
Drying step	Not required	SpeedVac evaporation to dryness
Derivatization	Not required	Methoxyamination (O-methoxylamine, 16 h, RT, dark) + silylation (BSTFA:TMCS, 1 h, 70°C)
Final preparation	Direct injection (45 µL + / 45 µL −)	Addition of heptane + internal standard (tricosane 10 ppm)
Injection volume	1–1.5 µL	1 µL
QC strategy	Pooled QC samples (n=5), blanks	Pooled QC samples (n=6), blanks

Table S4: Signal processing workflow for LC-MS data.

Step	Description	Software (version)
Feature extraction	Molecular Feature Extraction (MFE), ion selection, filtering	MassHunter Profinder (B.08.00)
Recursive extraction	rMFE to refine feature list and reduce false positives	MassHunter Profinder (B.08.00)
Alignment	RT tolerance (1% + 2 min), mass tolerance (20 ppm + 2 mDa)	MassHunter Profinder (B.08.00)
Filtering	Removal of low-quality features (noise, missing values, duplicates)	MassHunter Profinder (B.08.00)
Missing value imputation	k-nearest neighbors (kNN)	MATLAB (custom script)
QC filtering	Features with RSD < 30% retained	MATLAB (custom script)

Table S5: Signal processing workflow for GC-MS data.

Step	Description	Software (version)
Data inspection	TIC evaluation, internal standard check	MassHunter Unknown Analysis (B.7.00)
Data conversion	Formatting for quantitative analysis	MassHunter Workstation GC/MS (B.04.01)
Deconvolution	Peak deconvolution and feature detection	MassHunter Unknown Analysis (B.7.00)
Annotation	Spectral matching (Fiehn library, NIST, custom library)	Fiehn library (v2008), NIST (v2.2)
Alignment	Feature alignment across samples	MassProfiler Professional (B.12.1)
Quantification	Peak integration and target ion assignment	MassHunter Quantitative (B.07.0)
Missing value imputation	kNN algorithm	MATLAB (custom script)
Normalization	JIVE normalization to reduce batch effects	MATLAB (custom script)
QC filtering	Features with RSD < 30% retained	MATLAB (custom script)

Table S6: Primers used for quantitative RT-PCR analysis.

Target gene	*Forward primer*	*Reverse primer*
**36B4**	**AGATGCAGCAGATCCGCAT**	**GTTCTTGCCCATCAGCACC**
**18S**	**CGGCTACCACATCCAAGGAA**	**GTCGGAATTACCGCGGCT**
**ACC1**	**TGACAGACTGATCGCAGAGAAAG**	**TGGAGAGCCCCACACACA**
**B2M**	**ACTGATACATACGCCTGCAGAGTT**	**TCACATGTCTCGATCCCAGTAGA**
**E-cadherin**	**CACCTGGAGAGAGGCCATGT**	**TGGGAAACATGAGCAGCTCT**
**mt12S**	**TTGGTAAATTTCGTGGCAGCCACC**	**CAGTTTGGGTCTTAGCTGTCGTGT**
**mtCo1**	**CTCGCCTAATTTATTCCACTTCA**	**GGGGCTAGGGGTAGGGTTAT**
**mtCo2**	**ACCTGGTGAACTACGACTGCTAGA**	**TGCTTGATTTAGTCGGCCTGGGAT**
**mtCytB**	**ACCAATCTCCCAAACCATCA**	**TCCAGAGACTTGGGGATCTAAC**
**Mfn1**	**AATTAACTTCCTGCGTTGCTTT**	**GATGGAACCCACCAAACCCA**
**mtND1**	**GGGATAACAGCGCAATCCTA**	**ATCGTTGAACAAACGAACCA**
**N-cadherin**	**ATGTGCCGGATAGCGGGAGC**	**TACACCGTGCCGTCCTCGTC**
**OPA1**	**TGCCCTAACCATTTCAGAGGG**	**TCGAGAGCTCCCATCCCTAC**
**PGC1α**	AACCACACCCACAGGATCA	CTCTTCGCTTTATTGCTCCATGA
**PGC1β**	CTGACGTGGACGAGCTTTCA	CCTTCAGAGCGTCAGAGCTT
**PPARα**	**CCTCAGGGTACCACTAGGGAGT**	**GCCCGAATAGTTCGCCGAAA**
**TGFβ1**	**GGATACCAACTATTGCTTCAG**	**TGTCCAGGCTCCAAATATAG**
**TGFβ2**	**GAGATTTGCAGGTATTGATGG**	**CAACAACATTAGCAGGAGATG**
**TGFβ3**	**GGTTACTATGCCAACTTCTG**	**CACATAGTACAAGATGGTCAG**
**β-catenin**	**ACTGCTGGGACTCTG**	**TGATGGCGTAGAACAG**

Table S7: Antibodies used for immunohistochemistry and related analyses.

Target	Supplier	Reference	Dilution
Nitrotirosine	Millipore	06-284	1:100
α-SMA	Dako	M0851	1:100
CD36	Santa Cruz	sc7309	1:50
Anti-Mouse IgG	Bio-Rad	170-6516	1:5000
Anti-Rabbit IgG	Bio-Rad	170-6515	1:5000
